# Development of EST-based SNP and InDel markers and their utilization in tetraploid cotton genetic mapping

**DOI:** 10.1186/1471-2164-15-1046

**Published:** 2014-12-01

**Authors:** Ximei Li, Wenhui Gao, Huanle Guo, Xianlong Zhang, David D Fang, Zhongxu Lin

**Affiliations:** National Key Laboratory of Crop Genetic Improvement & National Centre of Plant Gene Research (Wuhan), Huazhong Agricultural University, Wuhan, 430070 Hubei China; College of Agronomy and Plant Protection, Qingdao Agricultural University/Shandong Key Laboratory of Dryland Farming Technology, Qingdao, 266109 Shandong China; Cotton Fiber Bioscience Research Unit, USDA–ARS, Southern Regional Research Center, New Orleans, LA 70124 USA

**Keywords:** Cotton, Molecular markers, SNP, InDel, Genetic mapping

## Abstract

**Background:**

Availability of molecular markers has proven to be an efficient tool in facilitating progress in plant breeding, which is particularly important in the case of less researched crops such as cotton. Considering the obvious advantages of single nucleotide polymorphisms (SNPs) and insertion-deletion polymorphisms (InDels), expressed sequence tags (ESTs) were analyzed *in silico* to identify SNPs and InDels in this study, aiming to develop more molecular markers in cotton.

**Results:**

A total of 1,349 EST-based SNP and InDel markers were developed by comparing ESTs between *Gossypium hirsutum* and *G. barbadense*, mining *G. hirsutum* unigenes, and analyzing 3′ untranslated region (3′UTR) sequences. The marker polymorphisms were investigated using the two parents of the mapping population based on the single-strand conformation polymorphism (SSCP) analysis. Of all the markers, 137 (10.16%) were polymorphic, and revealed 142 loci. Linkage analysis using a BC_1_ population mapped 133 loci on the 26 chromosomes. Statistical analysis of base variations in SNPs showed that base transitions accounted for 55.78% of the total base variations and gene ontology indicated that cotton genes varied greatly in harboring SNPs ranging from 1.00 to 24.00 SNPs per gene. Sanger sequencing of three randomly selected SNP markers revealed discrepancy between the *in silico* predicted sequences and the actual sequencing results.

**Conclusions:**

*In silico* analysis is a double-edged blade to develop EST-SNP/InDel markers. On the one hand, the designed markers can be well used in tetraploid cotton genetic mapping. And it plays a certain role in revealing transition preference and SNP frequency of cotton genes. On the other hand, the developmental efficiency of markers and polymorphism of designed primers are comparatively low.

**Electronic supplementary material:**

The online version of this article (doi:10.1186/1471-2164-15-1046) contains supplementary material, which is available to authorized users.

## Background

Molecular markers are the foundation of modern molecular plant breeding. There are many types of molecular markers such as restriction fragment length polymorphism (RFLP), simple sequence repeat (SSR) and SNPs. In cotton which is the world most important natural fiber crop, the most prevalent marker type as of today is SSR. However, with the advent of next generation sequencing technologies that significantly reduce sequencing cost, SNP markers are becoming more and more important due to their abundance in the genome and very simple genetic mode (bi-allelic).

Cotton researchers have tried different methods to develop SNP markers. An et al. [1] reported a few SNP markers when studying R2R3-MYB transcription factors. In 2009, Van Deynze [2] reported the first large-scale SNP discovery results in cotton. They developed about 1,000 SNPs and 300 InDels by re-sequencing the ESTs of 24 upland cotton genotypes. About 200 of these SNPs were also mapped in the TM-1 × 3-79 genetic map [3, 4]. Recently, research in cotton SNP discovery has been accelerated and many SNP markers have been reported [5, 6]. In spite of this, the number of cotton SNP markers is still low as compared with other major crops such as maize or soybean. More importantly, a great majority of these SNP markers have neither been validated in other genotypes nor mapped.

Due to its allotetraploid nature, it has been a challenge to differentiate a true SNP (within a sub-genome) from a pseudo-SNP (between subgenomes) in cotton SNP marker development. In 2009, Trick et al. [7] developed many SNP markers in *Brassica napus* that is also an allotetraploid using transcriptome sequencing. This study provided some insights that could be useful in the development of cotton SNP markers.

Direct sequencing has been a standard method to develop SNP markers, although its efficiency is low, especially in plants with complex genomes [8, 9]. Another alternative is to take advantage of the large amount of sequence data available in public databases to develop SNP and InDel markers using bioinformatics [10].

Expressed sequence tags (ESTs) have been mined for large-scale SNP discovery in plants including *Arabidopsis* [11], barley [12], maize [13], sugarcane [14], tomato [15], and cotton [2]. SNPs mined from ESTs have the potential to be functional markers if the particular EST or gene is responsible for phenotypic variations [16, 17]. Several methods for identifying SNPs from ESTs have been reported [17, 18], and numerous cotton ESTs are available in public databases [19], providing important foundations for the development of EST-based cotton SNP markers.

The 3′ untranslated regions (UTRs) undergo less selective pressure than the coding sequences (CDSs) [20], resulting in a higher rate of sequence variation than the CDSs. Thus, 3′UTRs have become valuable resources in identifying SNPs or InDels, especially in those species with duplicate genomes [21]. Koepke et al. [22] developed InDel markers by focusing on the 3′UTRs of the RNA-seq data in sweet cherry.

In the present study, cotton SNP and InDel markers were developed using four strategies. First, interspecific EST-SNPs were developed by comparing the ESTs between *G. hirsutum* and *G. barbadense.* Second, intraspecific EST-SNPs were developed by mining the unigenes of *G. hirsutum.* Third, EST-InDels were developed by mining the 3′UTRs of public *G. hirsutum* sequences. And fourth, InDel markers were developed by blasting putative 3′UTRs of *G. hirsutum* against the 3′UTRs of *Arabidopsis*. Subsequently, we used SSCP technology to validate these markers, and analyzed their polymorphisms between the two mapping parents. Polymorphic markers were used to genotype our BC_1_ mapping population [19] and mapped. SNP and InDel markers developed in this report will be a valuable genomic resource for cotton genetics and breeding research.

## Results

### *In silico*analysis and primer design

#### Interspecific EST-SNP markers

The collected 273,779 *G. hirsutum* ESTs and 11,311 *G. barbadense* ESTs were clustered into 3,263 clusters, which were then imported into HaploSNPer (http://www.bioinformatics.nl/tools/haplosnper/) to identify the interspecific SNPs. Of the 3,263 clusters, 1,668 (51.12%) had no SNPs, 109 (3.34%) had only inter-homoeologous SNPs, 200 (6.13%) had inter/hemi-SNPs, and 1,286 (39.41%) had hemi-SNPs. Only the clusters containing inter/hemi-SNPs or hemi-SNPs were used to design primers to detect interspecific SNPs (see Additional files 1 and 2).

Among the 200 clusters containing inter/hemi-SNPs, the number of clusters containing 4, 5, 6, 7 and ≥8 sequences was 46, 28, 24, 15 and 87, respectively. Five clusters were removed due to failing to meet the stringent criteria described in the ‘Methods’. Among the remaining 195 clusters, the number of clusters containing 4, 5, 6, 7 and ≥8 sequences were 43 (93.48%), 27 (96.43%), 23 (95.83%), 15 (100.00%) and 87 (100.00%), respectively. Eventually, 27, 21, 16, 11 and 59 inter/hemi-SNPs, and 0, 2, 1, 2 and 10 hemi-SNPs were developed, respectively (see Additional file 3). Detailed information of the markers, sequence accession numbers and sequences used to design primers, and SNPs of 134 inter/hemi-SNPs and 15 hemi-SNPs are listed in the Additional file 1.

To those 1,286 clusters containing only hemi-SNPs, the same classification analysis was conducted. After selection of the same stringent criteria, only 276 (21.46%) clusters were kept for SNP marker design. Finally, a total of 207 hemi-SNPs were developed (see Additional files 1 and 4).

#### Intraspecific EST-SNP markers

There were a total of 21,738 unigenes available from NCBI (http://www.ncbi.nlm.nih.gov/) in July 2010 when this research was initiated, derived from more than 19 cotton genotypes. After removing 7,449 unigenes containing less than 4 sequences, the remaining 14,289 unigenes were downloaded from NCBI, and the 1,339 unigenes containing sequences originating from the same genotype were further removed. As a result, 12,950 unigenes were imported into HaploSNPer to identify EST-SNPs. The results showed that 4,378 unigenes did not contain SNPs, while 8,572 unigenes contained SNPs.

The 8,572 unigenes were first classified according to the previously mentioned standards. Then the unigenes containing only putative SNPs were removed, and only 475 (5.54%) unigenes containing reliable SNPs were kept for primer design. Finally, 455 intraspecific EST-SNP markers were designed (see Additional files 5 and 6). In general, one primer pair was developed from each unigene. However, ideal primer pair could not be designed from some unigenes; and two or more primer pairs were designed from the unigenes containing some distant SNPs to amplify more possible SNPs.

#### InDel markers of G. hirsutum

A total of 8,938 *G. hirsutum* nucleiotide sequences were evaluated for possible InDel marker development, and 1,021 sequences with complete CDSs were selected. Among these sequences, 615 had 3′UTR sequences longer than 100 bp. After removing redundancy, 509 unique sequences were eligible to develop InDel markers. In total, 415 HAU-InDel-prefixed markers were developed (see Additional file 7).

Three sets of *G. hirsutum* sequences, including 65,520 genome survey sequences (GSS), 15,815 nucleotides sequences and 65,371 mRNAs were used to blast against the 25,843 3′UTRs of *Arabidopsis* sequences. According to the criteria described in the ‘Methods’, 8, 107 and 218 sequences were homologous to the 3’UTRs of *Arabidopsis* sequences including 111 singlets and 62 contigs. Subsequently, another 123 HAU-InDel-prefixed markers were developed (see Additional files 8 and 9).

### Polymorphisms of the SNP and InDel markers

All the 1,349 SNP and InDel markers described above were analyzed for their polymorphisms between *G. hirsutum* cv. Emian22 and *G. barbadense* acc. 3–79 using SSCP method. As a result, 137 (10.16%) primer pairs were polymorphic, and produced 142 loci (Table 1).

**Table 1:** **Polymorphic rates of the SNP and InDel markers**

Primers	No. markers	No. polymorphic markers/loci	Polymorphic rate (%)	Classes	Subclasses ^5)^	No. Markers	No. Polymorphic markers/loci	Polymorphic rate (%)
HAU-SNP^1)^	356	47/50	13.20		4	27	6/6	22.22
				inter	5	21	4/5	19.05
				23/134	6	16	2/3	12.50
				17.16%	7	11	0/0	0.00
					≥8	59	11/11	18.64
					4	0	0/0	--
				hemi	5	8	1/1	12.50
				24/222	6	9	2/2	22.22
				10.81%	7	10	0/0	0.00
					≥8	195	21/22	10.77
HAU-SNP^2)^	455	43/43	9.45		4	171	15/15	8.77
					5	89	12/12	13.48
					6	97	6/6	6.19
					7	38	3/3	7.89
					≥8	60	7/7	11.67
HAU-InDel^3)^	415	41/42	9.88					
HAU-InDel^4)^	123	6/7	4.88					
Total	1,349	137/142	10.16					

^1)^HAU-SNP001 ~ 356, which were developed by comparing ESTs between *G. hirsutum* and *G. barbadense*.

^2)^HAU-SNP357 ~ 811, which were developed by mining *G. hirsutum* unigenes.

^3)^HAU-InDel001 ~ 415, which were developed by mining the 3′UTRs of public *G. hirsutum* sequences.

^4)^HAU-InDel416 ~ 538, which were developed by blasting putative 3′UTRs of *G. hirsutum* against the 3′UTRs of *Arabidopsis*.

^5)^Subclasses mean different types of clusters/unigenes classified by the number of sequences contained in a cluster/unigene.

Among the 356 interspecific EST-SNP primer pairs, 47 (13.20%) pairs were polymorphic, and revealed 50 loci, including 23 inter/hemi-SNPs and 24 hemi-SNPs (Table 1). The 23 polymorphic inter/hemi-SNPs were screened out of 134 primer pairs, with a polymorphic rate of 17.16%. And the 24 polymorphic hemi-SNPs were selected from 222 primer pairs, with a polymorphic rate of 10.81%. SSCP analysis revealed 43 (9.45%) polymorphic markers out of the total 455 intraspecific EST-SNP primer pairs, producing 43 loci (Table 1). As for the polymorphisms of the InDel markers, 47 were polymorphic, and revealed 49 loci. More specifically, of the total 415 InDel markers designed through *G. hirsutum* mRNAs, 41 (9.88%) were polymorphic, and produced 42 loci (Table 1). Among the total 123 InDel markers developed from blast analysis against *Arabidopsis* 3′UTRs, 6 (4.88%) were polymorphic, and produced 7 loci (Table 1).

### Distribution of SNP and InDel markers on the interspecific BC_1_ linkage map

After linkage analysis, 133 of the 142 SNP and InDel polymorphic loci were mapped on the 26 cotton chromosomes. Sixty-six loci were mapped on the 13 chromosomes of the A_T_ genome, which included 1,204 loci with a total genetic distance of 2,297.27 cM and an average marker interval of 1.91 cM. Sixty-seven loci were mapped on the 13 chromosomes of the D_T_ genome, which included 1,415 loci with a total genetic distance of 2,246.24 cM and an average marker interval of 1.59 cM. The present interspecific linkage map contains 2,619 loci with a total genetic distance of 4,543.51 cM and an average marker interval of 1.73 cM (Table 2, and also see Additional file 10). Although the 133 SNP and InDel markers were mapped on all 26 chromosomes, they were not evenly distributed. Chr09, Chr10, Chr19 and Chr26 had more loci, while Chr04 and Chr06 had fewer loci (Table 2, see Additional file 10).

**Table 2:** **Distribution of SNP and InDel markers on the interspecific BC**_**1**_ **linkage map**

Chromosome	Size (cM)	Marker interval (cM)	Total loci	SNP loci	HAU-SNP loci ^1)^	HAU-SNP loci ^2)^	HAU-InDel loci ^3)^	HAU-InDel loci ^4)^
Chr01	186.87	2.46	76	3	1	2	0	0
Chr02	156.03	2.40	65	6	3	2	1	0
Chr03	156.23	2.06	76	5	2	2	1	0
Chr04	140.07	2.50	56	1	0	0	1	0
Chr05	242.76	1.75	139	6	1	2	3	0
Chr06	171.43	2.04	84	1	1	0	0	0
Chr07	103.39	1.48	70	4	2	2	0	0
Chr08	148.05	1.53	97	4	0	3	1	0
Chr09	148.83	1.43	104	9	2	3	3	1
Chr10	179.66	1.91	94	9	4	2	2	1
Chr11	234.77	1.63	144	7	1	2	4	0
Chr12	221.04	2.19	101	6	3	0	2	1
Chr13	208.14	2.12	98	5	2	2	1	0
A_T_ genome	2297.27	1.91	1204	66	22	22	19	3
Chr14	156.15	1.63	96	4	1	0	2	1
Chr15	189.00	1.60	118	5	2	0	3	0
Chr16	94.32	0.99	95	5	4	1	0	0
Chr17	149.43	2.13	70	5	3	2	0	0
Chr18	146.95	1.47	100	6	3	2	0	1
Chr19	252.27	1.64	154	8	1	4	3	0
Chr20	107.50	1.00	108	6	3	0	2	1
Chr21	256.03	1.82	141	5	1	3	1	0
Chr22	166.03	1.84	90	3	1	0	2	0
Chr23	193.19	1.82	106	4	1	2	1	0
Chr24	187.04	1.64	114	3	1	1	1	0
Chr25	151.25	1.41	107	5	3	1	1	0
Chr26	197.09	1.70	116	8	1	3	3	1
D_T_ genome	2246.24	1.59	1415	67	25	19	19	4
Total	4543.51	1.73	2619	133	47	41	38	7

^1)^HAU-SNP001 ~ 356.

^2)^HAU-SNP357 ~ 811.

^3)^HAU-InDel001 ~ 415.

^4)^HAU-InDel416 ~ 538.

### Statistical analysis of base variations and SNP frequency of cotton genes

Statistical analysis of reliable base variations showed that the SNPs in different clusters/unigenes that were used to design HAU-SNP-prefixed markers had the same tendency towards more base transitions (C → T or G → A) (Table 3). Specifically, the percentage of a certain base variation varied from 1.27% (C/-) to 28.56% (C → T) in the clusters used to design the interspecific EST-SNP markers, and from 2.30% (C/-) to 28.64% (C → T) in the unigenes used to design the intraspecific EST-SNP markers (Table 3). In total, the percentage varied from 1.67% (C/-) to 28.59% (C → T) in this study, with base transitions (C → T or G → A) accounting for 55.78% of the total SNPs (Table 3).

**Table 3:** **Summary of cotton base variations**

Types of SNPs	Interspecific EST-SNPs			Intraspecific EST-SNPs						Total
	Inter/hemi-SNPs	Hemi-SNPs	Subtotal	4	5	6	7	≥8	Subtotal	
C → T	265 (26.26%)	1036 (29.21%)	1301 (28.56%)	158 (30.10%)	153 (36.00%)	249 (27.04%)	103 (29.94%)	170 (24.50%)	833 (28.64%)	2134 (28.59%)
G → A	271(26.86%)	981 (27.66%)	1252 (27.48%)	147 (28.00%)	120 (28.24%)	244 (26.49%)	97 (28.20%)	170 (24.50%)	778 (26.74%)	2030 (27.19%)
All transitions	536 (53.12%)	2017 (56.86%)	2553 (56.04%)	305 (58.10%)	273 (64.24%)	493 (53.53%)	200 (58.14%)	340 (48.99%)	1611 (55.38%)	4164 (55.78%)
C → G	74 (7.33%)	282 (7.95%)	356 (7.81%)	40 (7.62%)	12 (2.82%)	61 (6.62%)	17 (4.94%)	47 (6.77%)	177 (6.09%)	533 (7.14%)
A → T	82 (8.13%)	410 (11.56%)	492 (10.80%)	45 (8.57%)	41 (9.65%)	96 (10.42%)	33 (9.59%)	61 (8.79%)	276 (9.49%)	768 (10.29%)
C → A	88 (8.72%)	304 (8.57%)	392 (8.60%)	33 (6.29%)	31 (7.29%)	67 (7.27%)	23 (6.69%)	53 (7.64%)	207 (7.12%)	599 (8.02%)
T → G	97 (9.61%)	304 (8.57%)	401 (8.80%)	42 (8.00%)	32 (7.53%)	79 (8.58%)	19 (5.52%)	73 (10.52%)	245 (8.42%)	646 (8.65%)
All transversions	341 (33.80%)	1300 (36.65%)	1641 (36.02)	160 (30.48%)	116 (27.29%)	303 (32.90%)	92 (26.74%)	234 (33.72%)	905 (31.11%)	2546 (34.11%)
A/-	52 (5.15%)	56 (1.58%)	108 (2.37%)	18 (3.43%)	13 (3.06%)	40 (4.34%)	25 (7.27%)	47 (6.77%)	143 (4.92%)	251 (3.36%)
C/-	22 (2.18%)	36 (1.01%)	58 (1.27%)	13 (2.48%)	5 (1.18%)	29 (3.15%)	4 (1.16%)	16 (2.31%)	67 (2.30%)	125 (1.67%)
G/-	28 (2.78%)	37 (1.04%)	65 (1.43%)	16 (3.05%)	3 (0.71%)	27 (2.93%)	11 (3.20%)	22 (3.17%)	79 (2.72%)	144 (1.93%)
T/-	30 (2.97%)	101 (2.85%)	131 (2.88%)	13 (2.48%)	15 (3.53%)	29 (3.15%)	12 (3.49%)	35 (5.04%)	104 (3.58%)	235 (3.15%)
All InDels	132 (13.08%)	230 (6.48%)	362 (7.95%)	60 (11.43%)	36 (8.47%)	125 (13.57%)	52 (15.12%)	120 (17.29%)	393 (13.51%)	755 (10.11%)
Total	1009 (100.00%)	3547 (100.00%)	4556 (100.00%)	525 (100.00%)	425 (100.00%)	921 (100.00%)	344 (100.00%)	694 (100.00%)	2909 (100.00%)	7465 (100.00%)

In order to gain more understanding about the relationship between SNPs and gene functions, we conducted functional annotation analyses of the consensus sequences. The number of SNPs/gene involved in cellular component, molecular function and biological process was 13.90, 9.36 and 10.56 respectively for those clusters used to design interspecific EST-SNP markers. In the unigenes used to design the intraspecific EST-SNP markers, a similar analysis showed that the number of SNPs/gene in cellular component, molecular function and biological process was 6.14, 6.46 and 5.21, respectively. In total, the highest number of SNPs/gene was in the cellular component category (11.96), followed by the molecular function (8.02) and biological process (7.92) categories (Table 4).

**Table 4:** **GO analysis of consensus sequences used to design the HAU-SNP-prefixed markers on level 1**

	Functional categories	Number of genes	Number of SNPs	SNPs/gene
**HAU-SNP** ^**1)**^	Cellular component	21	292	13.9
	Molecular function	107	1001	9.36
	Biological process	80	845	10.56
**HAU-SNP** ^**2)**^	Cellular component	7	43	6.14
	Molecular function	92	594	6.46
	Biological process	78	406	5.21
**Total**	Cellular component	28	335	11.96
	Molecular function	199	1595	8.02
	Biological process	158	1251	7.92

^1)^HAU-SNP001 ~ 356. ^2)^HAU-SNP357 ~ 811.

GO analysis of the total 947 consensus sequences on level 3 showed that 28 sequences were assigned to 4 functions in the ‘cellular component’ category, 199 were assigned to 24 functions in the ‘molecular function’ category, and 158 were assigned to 32 functions in the ‘biological process’ category (see Additional file 11). Among these functions, genes belonging to ‘killing of cells of other organism’ had the maximum SNPs/gene (24.00); while, genes belonging to ‘selenium binding’, ‘circadian rhythm’, etc. harbored the minimum SNPs/gene (1.00) (see Additional file 11).

### Confirmation of the predicted SNPs

**Figure 1:**
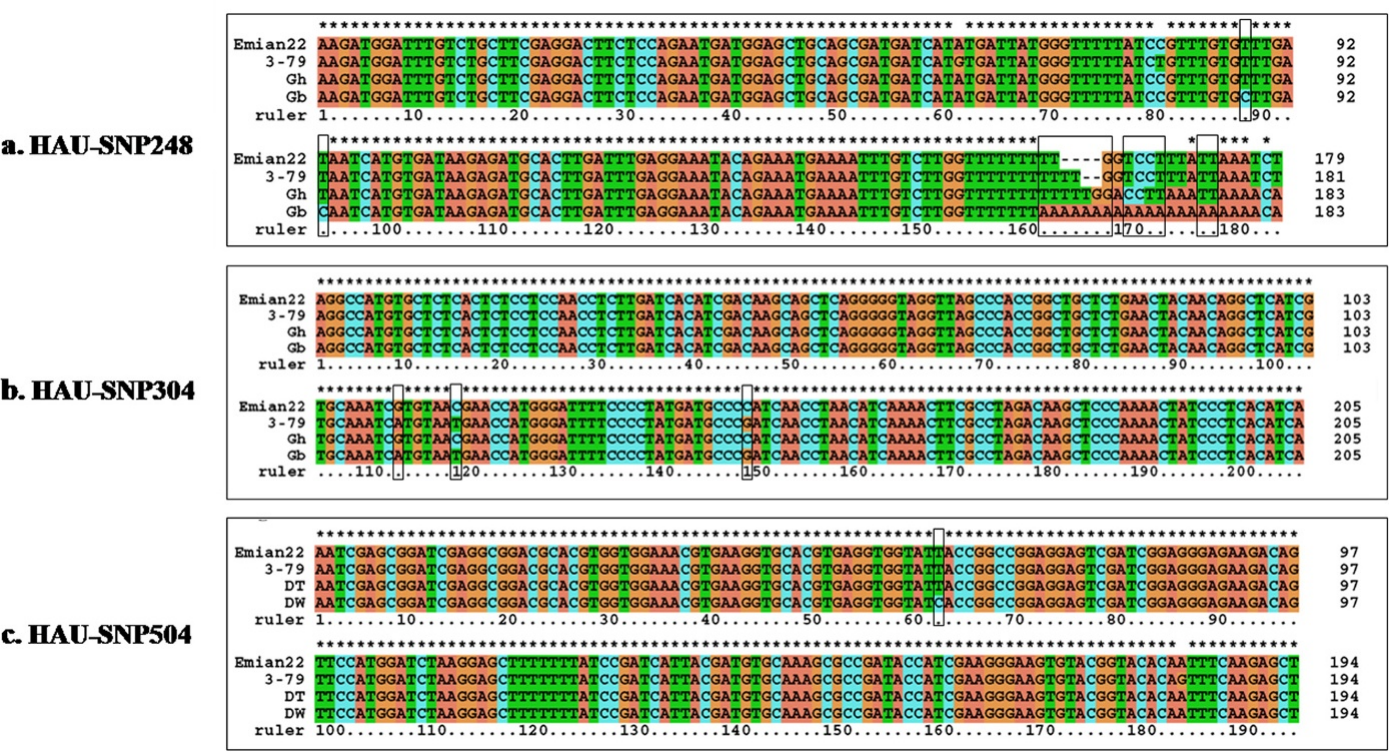
**Sequence comparisons between** ***in silico*** **analyses and actual Sanger-sequencing results of PCR products from two cotton genotypes (Emian 22 and 3–79).** Vector and primer sequences are removed, and the predicted SNPs are shown in boxes; Gh, Gb, DT and DW are from GenBank. **a)** Marker HAU-SNP248; **b)** Marker HAU-SNP304; and **c)** Marker HAU-SNP504.

To validate the SNPs predicted by *in silico* analysis, the PCR products generated from three polymorphic primer pairs were randomly chosen to be cloned and Sanger-sequenced. The results showed that the product sizes of two markers (HAU-SNP304 and HAU-SNP504) were not different between Emian22, 3–79 and the original sequences, but those of marker HAU-SNP248 were slightly different between them (Figure 1).

*In silico* analysis predicted several SNPs in the amplified products of the marker HAU-SNP248, including base transitions at the 88^th^ and 93^rd^ bases, and a series of base variations starting at the 161^st^ base. However, sequence analysis of the two mapping parents differed from the predicted results. Two base transitions appeared at the 61^st^ base (A in Emian22 → G in 3–79) and the 80^th^ base (C in Emian22 → T in 3–79). The interspecific differences in the end of the amplified sequences appeared at the 163^rd^ and 164^th^ bases (- in Emian22 → T in 3–79) (Figure 1a).

The three predicted interspecific SNPs in marker HAU-SNP304 included base transitions at the 112^nd^ and 118^th^ bases, and a base transversion at the 148^th^ base. These SNPs were confirmed by the two mapping parents without any discrepancy (Figure 1b).

Only one SNP was predicted in the amplified products of the marker HAU-SNP504, which was a transition at the 62^nd^ base (C in DW → T in DT). However, both Emian22 and 3–79 contained T at the 62^nd^ base, while the difference between the two parents appeared at the 183^rd^ base (A in Emian22 → G in 3–79). Besides the base transitions at the 62^nd^ and 183^rd^ bases, there was no difference among the four sequences (Figure 1c).

## Discussion

### Reliability of SNPs contained in clusters/unigenes

The *in silico* analysis of clusters used to design the interspecific EST-SNPs showed that, among the 200 clusters containing inter/hemi-SNPs, 195 (97.50%) contained reliable SNPs. This value increased with the number of sequences contained in the clusters increased (see Additional file 3). Similar trend was observed in the 1,286 clusters containing hemi-SNPs though the number of reliable SNPs was smaller (see Additional file 4). The reason behind this might be that most of the clusters containing hemi-SNPs had fewer *G. barbadense* sequences while more *G. barbadense* sequences existed for those containing inter/hemi-SNPs.

*In silico* analysis of unigenes used to design the intraspecific EST-SNPs showed that, only 475 (5.54%) of the total 8,572 unigenes containing putative SNPs harbored reliable SNPs. This number further decreased with the increasing number of sequences contained in the unigenes, ranging from 62.03% to 0.95% (see Additional file 6). This might be partially due to different sequences of the same genotype submitted by different researchers, along with the increase of the total number of sequences contained in the unigenes that demands more rigorous comparison.

### Polymorphism comparison of SNP and InDel markers

The polymorphic rate of the intraspecific EST-SNP markers (9.45%) was relatively low, which is consistent with the fact that markers derived from coding sequences have a lower polymorphism due to their more conserved nature compared to non-coding sequences [23]. The interspecific EST-SNP markers had the highest polymorphic rate (13.20%), which is mainly due to the focused attention on the interspecific differences while developing markers [7]. Although various polymorphic rates of markers among the subclasses existed in the both categories, the polymorphic rate of inter/hemi-SNPs (17.16%) was much higher than that of hemi-SNPs (10.81%).

Compared to the intraspecific EST-SNP markers, the first batch of HAU-InDel-prefixed markers had a higher polymorphic rate (9.88%), which is consistent with the results obtained by Zhu et al. [8]. However, the second batch of HAU-InDel-prefixed markers showed the lowest polymorphic rate (4.88%). Generally, this is a possible but unsatisfactory method to develop cotton markers by comparing with *Arabidopsis* sequences. Application of this method might result in inaccurate information. This inaccuracy may be overcome when tetraploid cotton genome sequence becomes available. In summary, appropriate methods of marker development, either through improving the reliability of predicted SNPs or targeting regions possessing more variations, are necessary to increase polymorphic rates.

### Even distribution of SNP and InDel markers on the interspecific BC_1_ linkage map

No obvious difference was observed in the number of SNP/InDel loci mapped between the A_T_ and D_T_ sub-genomes. However, uneven distribution was present among chromosomes. Within a chromosome, the SNP/InDel loci were relatively evenly distributed (Table 2, see Additional file 10). The results are consistent with the fact that base substitutions exist throughout the cotton genome [24].

### Transition preference and SNP frequency of cotton genes

Statistical analysis of the cotton base variations showed that base transitions appeared more frequently than other base variations, consistent with previous reports of the preference for base transitions [13, 25, 26]. Specifically, base transitions accounted for 55.78% of all the reliable SNPs in this study, which may be due to methylated cytosines in CpG dinucleotides changed into thymines during the genesis of the SNPs [27].

SNP frequency of cotton genes on level 1 showed that SNPs/gene decreased gradually in the order of cellular component category, molecular function category and biological process category (Table 4). On level 3, SNP frequency in each gene varied from 1.00 to 24.00 among genes with different functions (see Additional file 11). These results could provide directions to the research on SNP effects on gene functions.

### Advantages and disadvantages of developing EST-SNP/InDel markers

With the availability of large number of ESTs and the release of plant genomes, a large number of SNPs/InDels in various plants have been discovered using bioinformatics [11, 13, 15], indicating that bioinformatics is an efficient tool to discover SNPs/InDels. In this study, however, there were some differences between the predicted results and the sequencing results (Figure 1). The materials used for sequencing in our study are different from those materials for predicting SNPs/InDels, which may account for the differences observed. Additionally, the deviation between bioinformatics and experiments partly explained why these markers had such low polymorphism in this study. In conclusion, direct sequencing of the mapping parents may be the best way to develop highly reliable and polymorphic SNP/InDel markers.

## Conclusions

A total of 1,349 SNP/InDel markers were developed from a large number of ESTs. Of them, 137 markers (10.16%) were polymorphic between two mapping parents and revealed 142 polymorphic loci based on the SSCP analysis. Although the marker discovery efficiency and marker polymorphism were relatively low, linkage analysis mapped 133 loci on the 26 chromosomes, indicating that EST-based SNPs and InDels developed by *in silico* analysis are useful in tetraploid cotton genetic mapping. In addition, this study also revealed the preference of base transitions over other types of base variations and different SNP frequencies contained in cotton genes. Sanger sequencing showed certain discrepancy between the *in silico* sequence prediction and the actual sequences. In general, the *in silico* analysis is a complementary but of low efficiency method to develop SNPs and InDels in cotton, indicating that resequencing or high-throughput sequencing may be a better way to develop cotton SNPs/InDels.

## Methods

### Plant materials

*Gossypium hirsutum* cv. Emian22 and *G. barbadense* acc. 3–79 were used to detect polymorphisms of the newly developed SNP and InDel markers. The BC_1_ population [(Emian22 × 3-79) × Emian22] with 141 progenies [19] was used as the mapping population to map all polymorphic markers.

### *In silico*analysis and primer design

#### Interspecific EST-SNP markers

**Figure 2:**
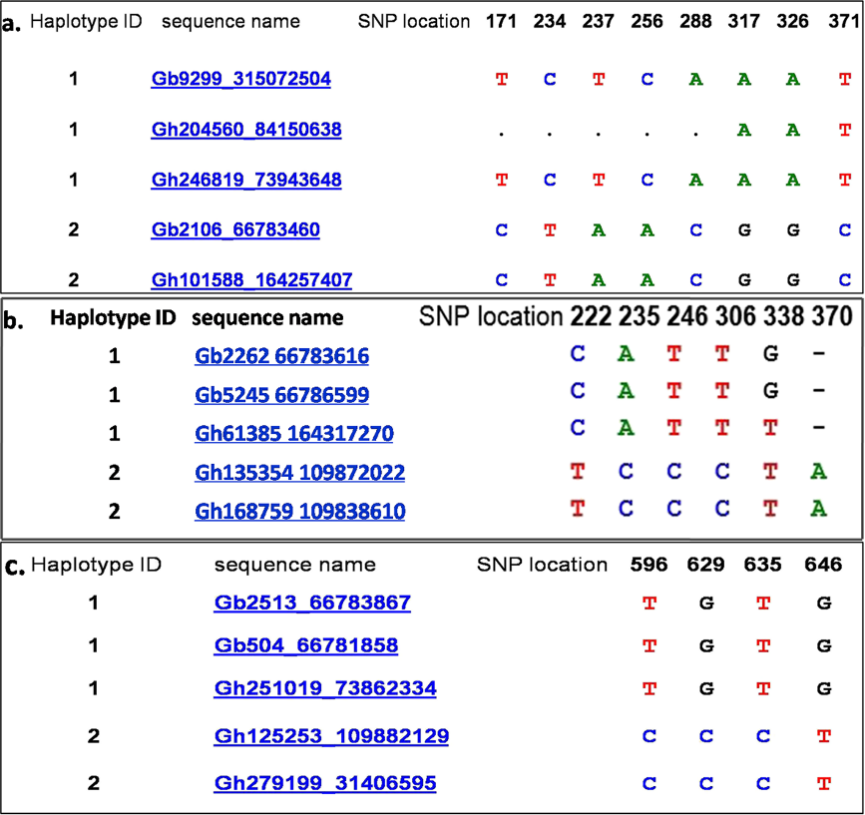
**Three types of clusters during** ***in silico*** **analysis of interspecific EST-SNPs. a**: Clusters with only inter-homoeologue SNPs (Both *G. hirsutum* and *G. barbadense* harbor two base types at a certain base, and no difference in base types exists between them); **b**: Clusters with inter/hemi-SNPs (*G. hirsutum* and *G. barbadense* harbor different base types at one or more bases); **c**: Clusters with only hemi-SNPs (One of *G. hirsutum* and *G. barbadense* harbors only one base type at a certain base, and the other one harbors two base types at the certain base. Base types between them are partially different).

The ESTs of *G. hirsutum* and *G. barbadense* downloaded from NCBI (http://www.ncbi.nlm.nih.gov/) were clustered using the wcd program [28]. All of the clusters were then imported into HaploSNPer (http://www.bioinformatics.nl/tools/haplosnper/) to identify SNPs between *G. hirsutum* and *G. barbadense* with default parameters. In this step, the clusters containing no SNPs or inter-homoeologous SNPs (Figure 2a) were discarded, while the clusters containing inter/hemi-SNPs (Figure 2b) or hemi-SNPs (Figure 2c) were kept. To be qualified for primer design, the clusters must contain two or more sequences from *G. hirsutum* and *G. barbadense*, respectively. Primer 3 (version 0.4.0) (http://frodo.wi.mit.edu/primer3/) was used to design primers with criteria as follows: primer length 18–24 bp, optimum 20 bp; GC content 40-60%, optimum 50%; optimum annealing temperature 58°C; and PCR product size 100–300 bp. The SNP markers developed were named as HAU-SNP001 ~ HAU-SNP356.

#### Intraspecific EST-SNP markers

**Figure 3:**
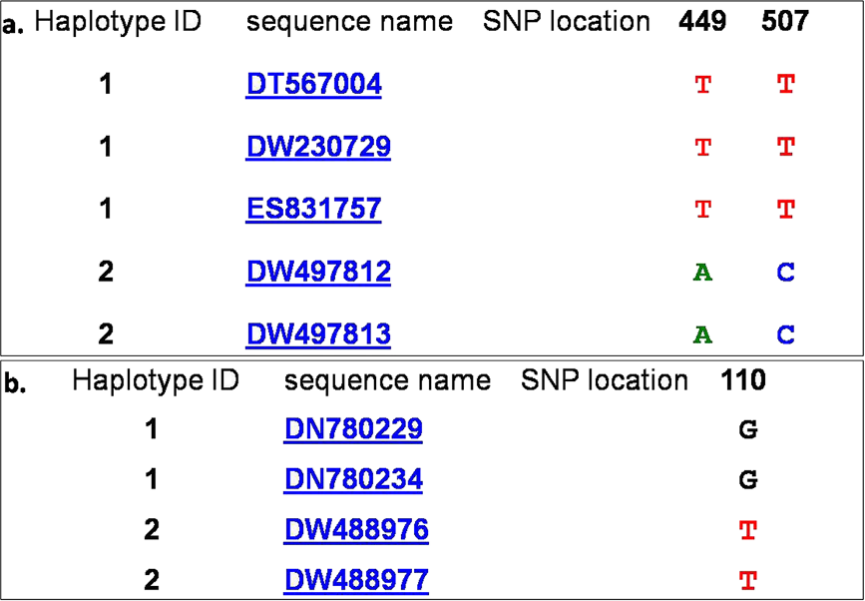
**Two types of unigenes during** ***in silico*** **analysis of intraspecific EST-SNPs. a**: Unigenes with only putative SNPs; **b**: Unigenes with reliable SNPs.

*G. hirsutum* unigenes with at least four ESTs were downloaded from NCBI, and then unigenes with ESTs from the same genotype were discarded. For the remaining unigenes, HaploSNPer was used to identify SNPs with default parameters. Unigenes that contained only potential SNPs predicted by HaploSNPer were further removed (Figure 3a), while unigenes containing reliable SNPs (Figure 3b) were used to design primers with criteria same as those of the interspecific EST-SNP primers. The SNP markers developed herein were named as HAU-SNP357 ~ HAU-SNP811.

#### InDel markers of G. hirsutum

Message RNA sequences with complete CDSs were selected from the collected *G. hirsutum* nucleiotide sequences. After removing redundancy, the remaining unique sequences that had 3′UTR sequences longer than 100 bp were used to develop HAU-InDel-prefixed markers to amplify InDels existing in the 3′UTRs. The criteria for primer design using Primer Premier 6.0 software (http://www.premierbiosoft.com) were as follows: primer length 18–24 bp, optimum 20 bp; GC content 35-60%, optimum 50%; optimum annealing temperature 55°C; and PCR product size 100–500 bp [29].

The 3′UTR sequences of *Arabidopsis* were kindly provided by Prof. Graziano Pesole (graziano.pesole@biologia.uniba.it). The collected genome survey sequences, nucleotide sequences and mRNA sequences of *G. hirsutum* were used to blast against the *Arabidopsis* 3′UTR sequences with E value of 1.0 × E^-10^ and matched sequence length of ≥100 bp. Subsequently, the redundant sequences were removed manually. The matched parts of the *G. hirsutum* sequences were also used to design HAU-InDel-prefixed primers following the similar methods described above.

### Genotyping markers using SSCP analysis

PCR amplification of SNP and InDel markers was conducted according to the methods described by Lin et al. [30]. Polymorphism detection between the two mapping parents and genotyping of the whole mapping population using polymorphic markers were carried out according to the improved SSCP technology described by Li et al. [31]. In brief, the amplified products were denatured in a boiling water bath for five minutes. The single-stranded DNA was separated on an 8% native polyacrylamide gel (29 acrylamide: 1 N,N-methylene bisacrylamide) at a constant watt of 15 W for about 4 h, and DNA fragments were detected with silver staining (an example see Additional file 12).

### Genetic mapping

The polymorphic SNP and InDel markers were integrated into our previously published interspecific BC_1_ linkage map [19, 29, 31, 32]. The logarithm of odds (LOD) threshold during map construction was 8.0, while the other parameters were the same as those described by Li et al. [31].

### Statistical analysis of base variations and SNP frequency of cotton genes

All the reliable SNPs were subjected to the statistical analysis, producing six kinds of base variations including A/- or T/-, C/- or G/-, A → G or T → C, A → C or T → G, A → T or T → A and C → G or G → C. In addition, the SNP frequency in cotton genes was evaluated by combining the gene ontology analyses of all the sequences used to design the HAU-SNP-prefixed markers. The functional annotation of nucleotide sequences was performed using Blast2GO [33, 34] with default parameters, and the subsequent analyses were conducted according to the methods described by Li et al. and annotations on level 3 were directly used [35].

### Validation of predicted SNPs using Sanger sequencing

The PCR-amplified products of randomly chosen SNP markers were recovered from agarose gels and purified using QIAGEN purification kits (QIAGEN, Dusseldorf, Germany). The purified amplicons were cloned into T-Easy vector (Promega, Madison, Wis., USA). Then at least three clones were randomly selected to be commercially sequenced from both ends using M13F and M13R primers. All above experimental procedures were according to the methods described by Li et al. [31]. After removing the vector and primer sequences, CLUSTAL_X [36] was used to compare the DNA sequences of the two parents and the original sequences.

## Electronic supplementary material

Additional file 1: **Details of the 356 interspecific EST-SNP markers.** SNP primer names, forward and reverse primer sequences, reference sequences used to design primers, and details of the interspecific SNPs are all listed. (XLS 2 MB)

Additional file 2: **Primary screening process of clusters used to develop interspecific EST-SNP markers.** Four types of clusters produced after identification of interspecific SNPs using HaploSNPer. (TIFF 99 KB)

Additional file 3: **Flowchart of developing inter/hemi-SNPs.** i: Primers amplifying inter/hemi-SNPs; h: Primers amplifying only hemi-SNPs. One hundred and thirty-four primers amplifying inter/hemi-SNPs, and 15 primers amplifying only hemi-SNPs were developed finally. (TIFF 172 KB)

Additional file 4: **Flowchart of developing hemi-SNPs.** h: Primers amplifying only hemi-SNPs. Two hundred and seven primers amplifying only hemi-SNPs were developed finally. (TIFF 169 KB)

Additional file 5: **Details of the 455 intraspecific EST-SNP markers.** SNP primer names, forward and reverse primer sequences, unigenes and reference sequences used to design primers, and details of the intraspecific SNPs are all listed. (XLS 757 KB)

Additional file 6: **Flowchart of developing intraspecific EST-SNP markers.** After several steps of selection, only 8,572 of the total 21,738 unigenes were eligible for further analysis to design intraspecific EST-SNP markers, and 455 markers amplifying intraspecific EST-SNPs were developed finally. (TIFF 244 KB)

Additional file 7: **Details of the 415 EST-InDel markers developed by mining the 3′UTRs of public** ***G. hirsutum*** **sequences.** InDel primer names, forward and reverse primer sequences, reference sequences used to design primers and their Genebank numbers are all listed. (XLS 713 KB)

Additional file 8: **Details of the 123 EST-InDel markers developed by blasting putative 3′UTRs of** ***G. hirsutum*** **against the 3′UTRs of** ***Arabidopsi.s*** InDel primer names, forward and reverse primer sequences, and reference sequences used to design primers are all listed. (XLS 80 KB)

Additional file 9: **Flowchart of developing HAU-InDel-prefixed markers by blasting putative 3′UTRs of** ***G. hirsutum*** **against the 3′UTRs of** ***Arabidopsis.*** Three parts of cotton 3′UTRs were undergone blast analysis against the *Arabidopsis* 3′UTRs respectively. Obtained unique sequences produced 62 contigs and 111 singlets, then 123 primers amplifying cotton InDels existing in 3′UTRs were developed. (TIFF 150 KB)

Additional file 10: **Linkage map of 26 cotton chromosomes based on an interspecific BC**_**1**_ **population.** SNP and InDel markers reported in this research are italicized, underlined and bolded. (PDF 10 MB)

Additional file 11: **SNP frequencies of cotton genes on level 3.** There were 4 sub-categories in cell component category **(a)**, 24 in molecular function category **(b)** and 32 in biological process category **(c)**. Among all the sub-categories, the number of SNPs/genes varied from 1.00 to 24.00. (TIFF 245 KB)

Additional file 12: **The electrophoresis gel of marker HAU-SNP572.** (TIFF 2 MB)
