# Supplementary figures and images for: Development of EST-based SNP and InDel markers and their utilization in tetraploid cotton genetic mapping
Source: BMC Genomics. 2014 Dec 1;15(1):1046. doi: 10.1186/1471-2164-15-1046 (PMC4265408; doi:10.1186/1471-2164-15-1046)

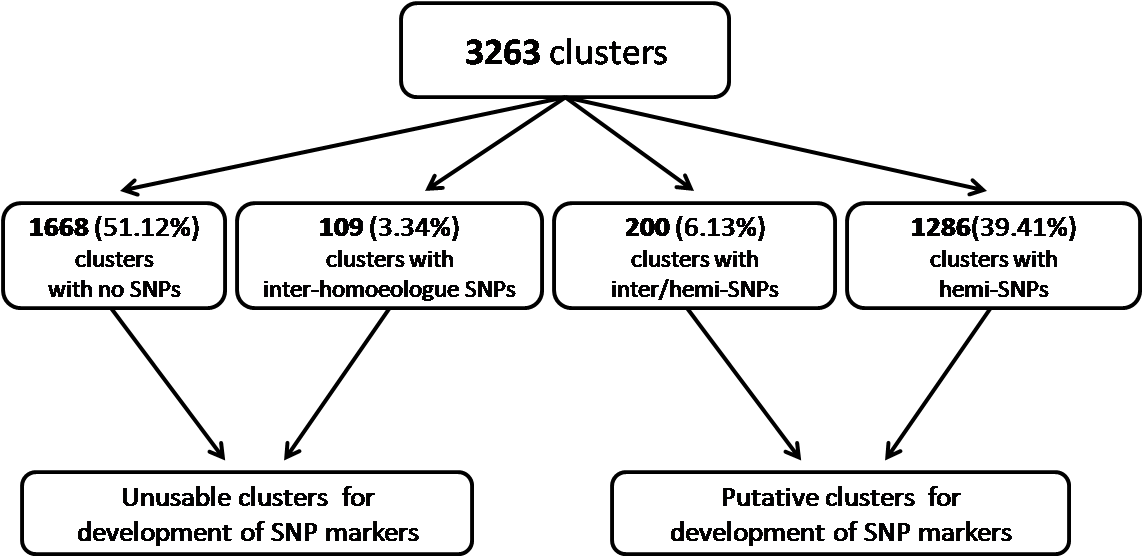

Supplement: Supplementary file 2 — Additional file 2: Primary screening process of clusters used to develop interspecific EST-SNP markers. Four types of clusters produced after identification of interspecific SNPs using HaploSNPer. (TIFF 99 KB) [file 12864_2014_6749_MOESM2_ESM.tiff]

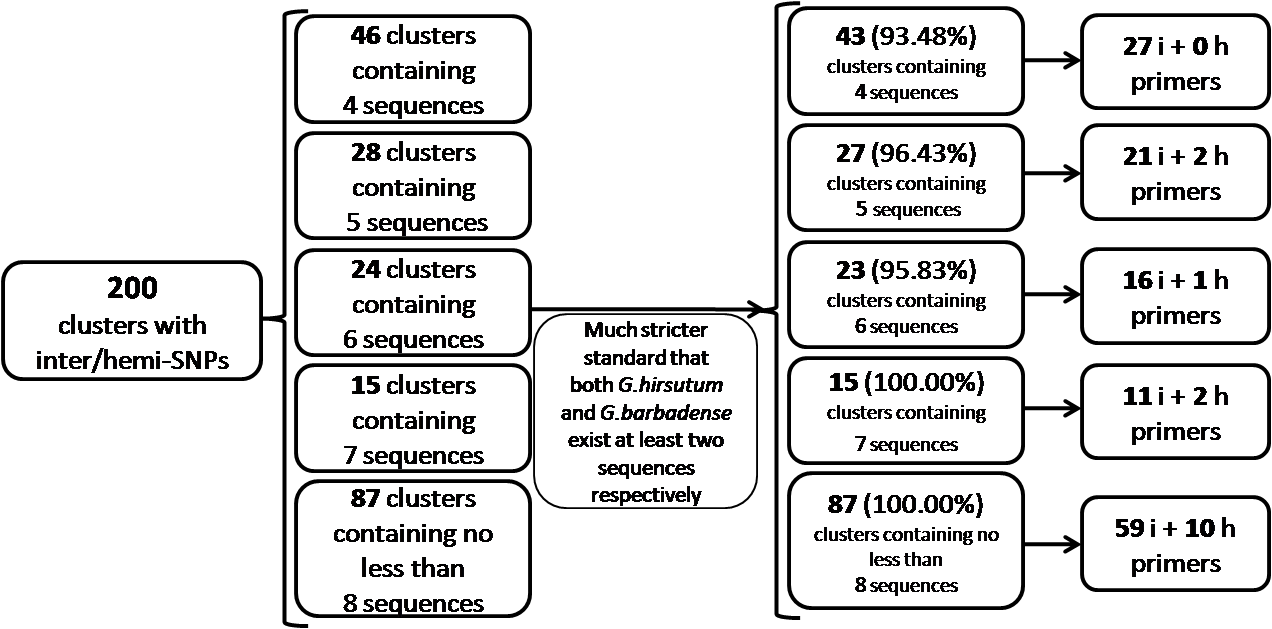

Supplement: Supplementary file 3 — Additional file 3: Flowchart of developing inter/hemi-SNPs. i: Primers amplifying inter/hemi-SNPs; h: Primers amplifying only hemi-SNPs. One hundred and thirty-four primers amplifying inter/hemi-SNPs, and 15 primers amplifying only hemi-SNPs were developed finally. (TIFF 172 KB) [file 12864_2014_6749_MOESM3_ESM.tiff]

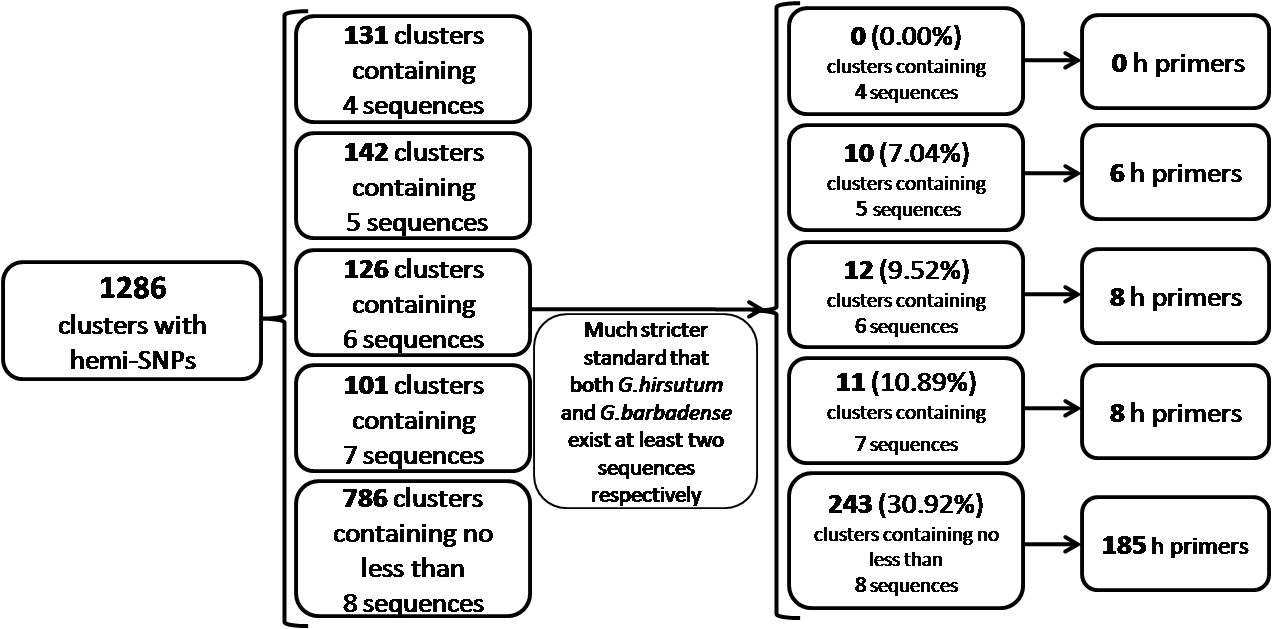

Supplement: Supplementary file 4 — Additional file 4: Flowchart of developing hemi-SNPs. h: Primers amplifying only hemi-SNPs. Two hundred and seven primers amplifying only hemi-SNPs were developed finally. (TIFF 169 KB) [file 12864_2014_6749_MOESM4_ESM.tiff]

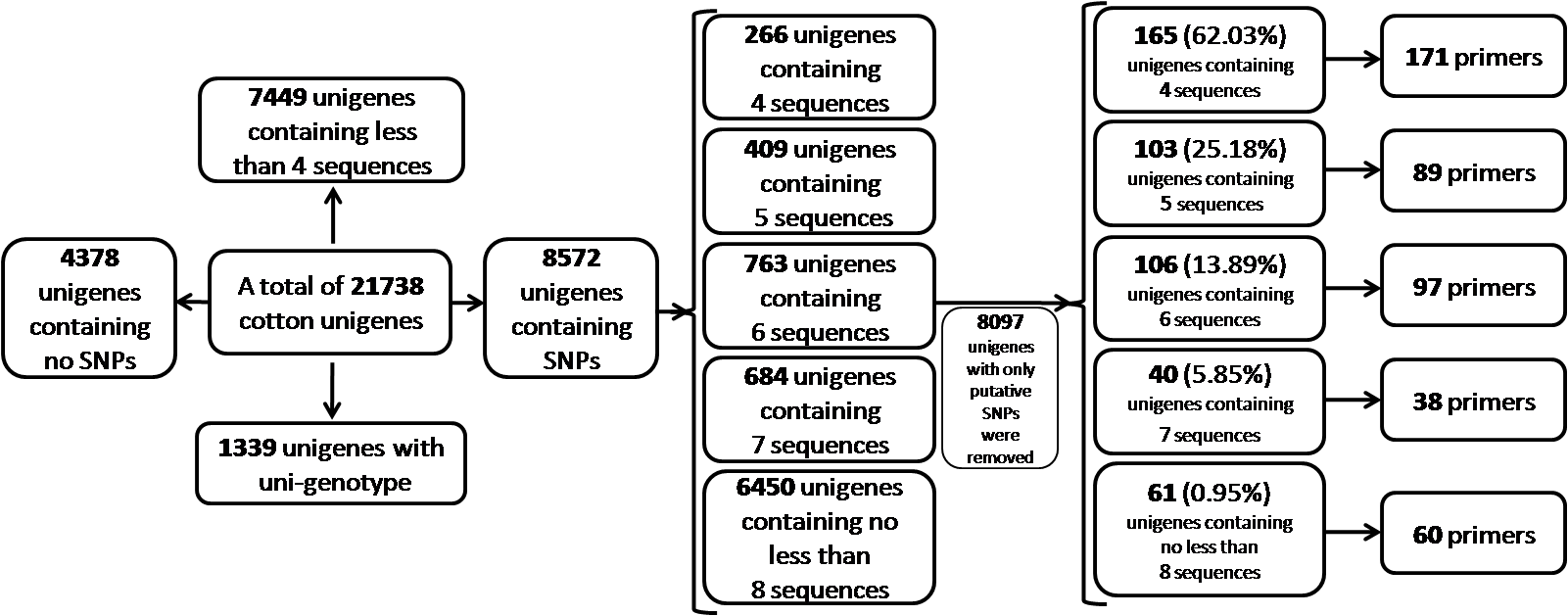

Supplement: Supplementary file 6 — Additional file 6: Flowchart of developing intraspecific EST-SNP markers. After several steps of selection, only 8,572 of the total 21,738 unigenes were eligible for further analysis to design intraspecific EST-SNP markers, and 455 markers amplifying intraspecific EST-SNPs were developed finally. (TIFF 244 KB) [file 12864_2014_6749_MOESM6_ESM.tiff]

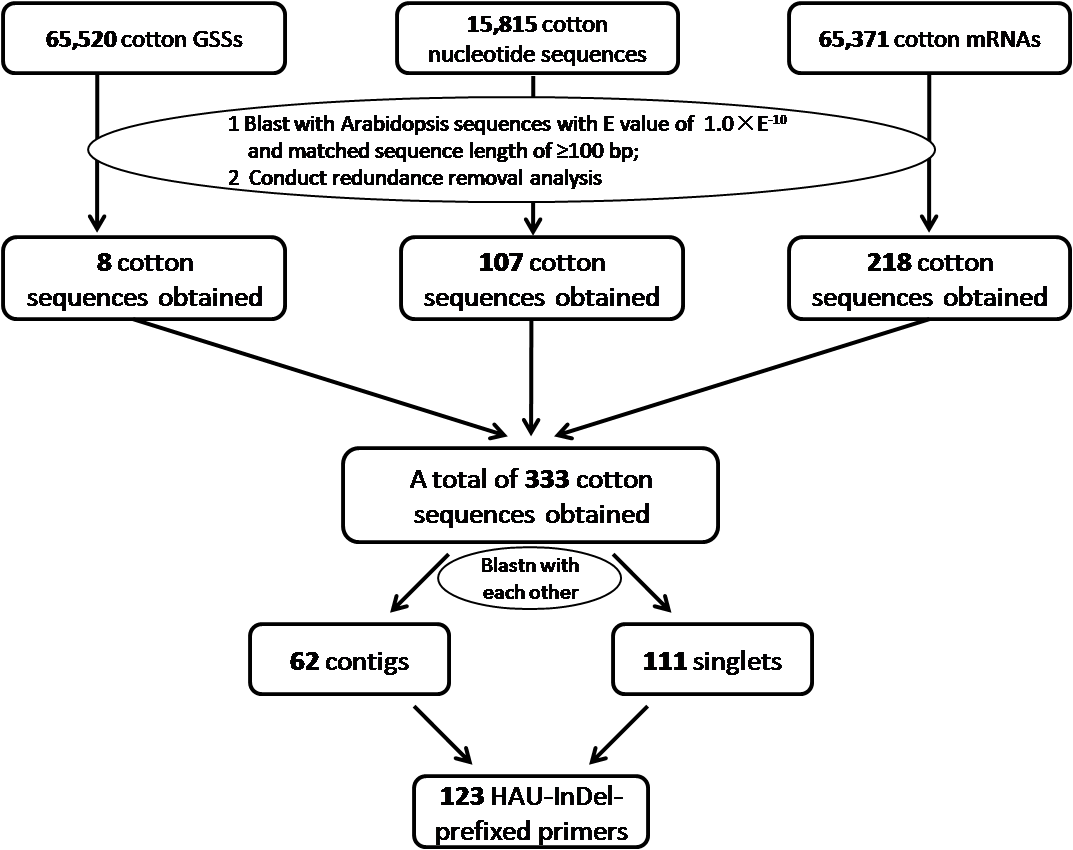

Supplement: Supplementary file 9 — Additional file 9: Flowchart of developing HAU-InDel-prefixed markers by blasting putative 3′UTRs of G. hirsutum against the 3′UTRs of Arabidopsis. Three parts of cotton 3′UTRs were undergone blast analysis against the Arabidopsis 3′UTRs respectively. Obtained unique sequences produced 62 contigs and 111 singlets, then 123 primers amplifying cotton InDels existing in 3′UTRs were developed. (TIFF 150 KB) [file 12864_2014_6749_MOESM9_ESM.tiff]

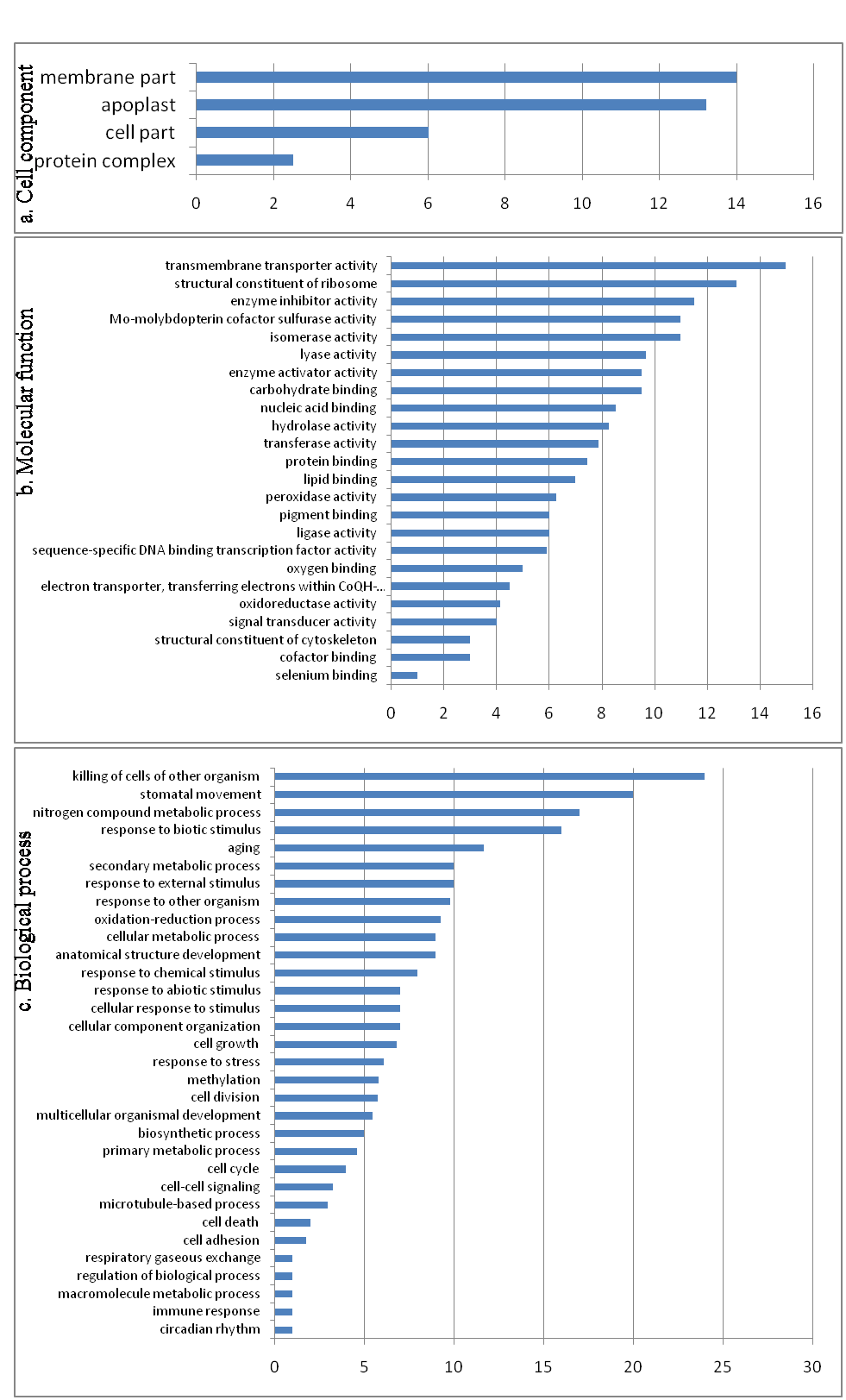

Supplement: Supplementary file 11 — Additional file 11: SNP frequencies of cotton genes on level 3. There were 4 sub-categories in cell component category (a), 24 in molecular function category (b) and 32 in biological process category (c). Among all the sub-categories, the number of SNPs/genes varied from 1.00 to 24.00. (TIFF 245 KB) [file 12864_2014_6749_MOESM11_ESM.tiff]

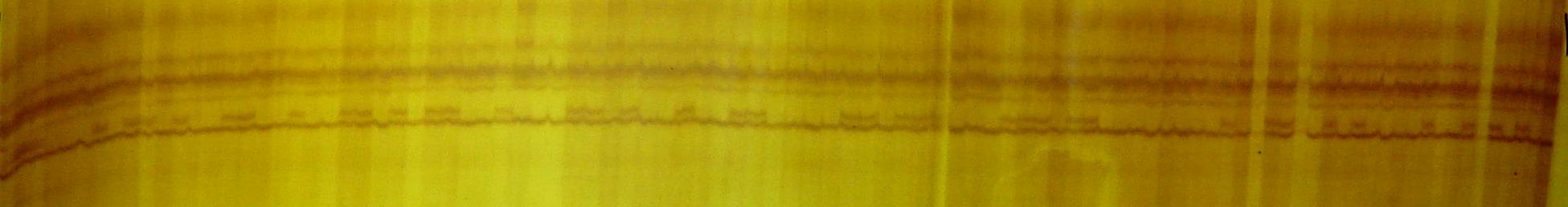

Supplement: Supplementary file 12 — Additional file 12: The electrophoresis gel of marker HAU-SNP572. (TIFF 2 MB) [file 12864_2014_6749_MOESM12_ESM.tiff]
