# Supplementary material for: Development of EST-based SNP and InDel markers and their utilization in tetraploid cotton genetic mapping
Source: BMC Genomics. 2014 Dec 1;15(1):1046. doi: 10.1186/1471-2164-15-1046 (PMC4265408; doi:10.1186/1471-2164-15-1046)
Supplement: Supplementary file 10 — Additional file 10: Linkage map of 26 cotton chromosomes based on an interspecific BC 1 population. SNP and InDel markers reported in this research are italicized, underlined and bolded. (PDF 10 MB) [file 12864_2014_6749_MOESM10_ESM.pdf]

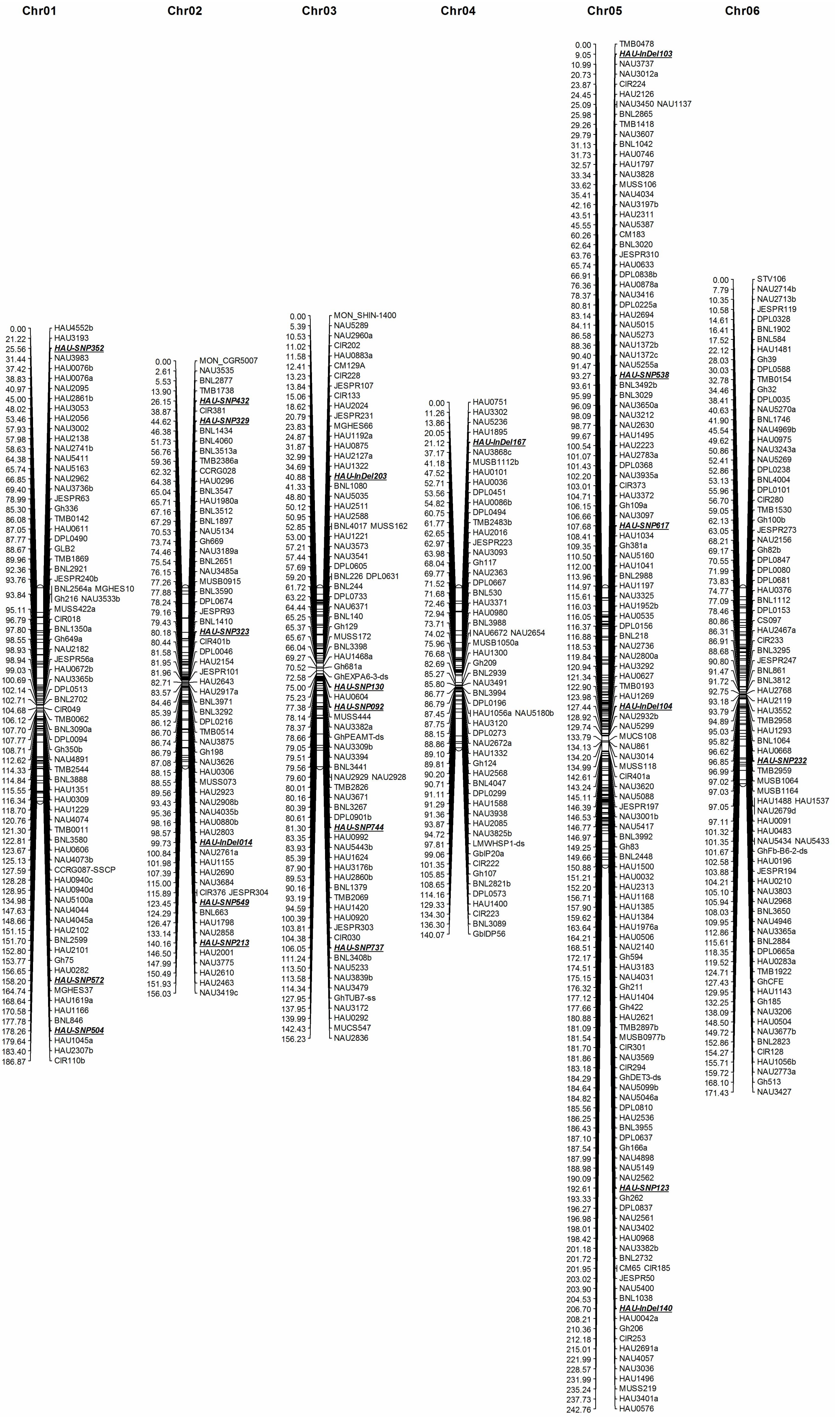
Chr01
Chr02
Chr03
Chr04
Chr05
Chr06
0.00 HAU4552b
21.22 HAU3193
25.56 HAU-SNP352
31.44 NAU3983
37.42 HAU0076b
38.83 HAU0076a
40.97 NAU2095
45.00 HAU2861b
48.02 HAU3053
53.46 HAU2056
57.93 NAU3002
57.98 NAU2138
58.63 NAU2741b
64.38 NAU5411
65.74 NAU5163
66.85 NAU2962
69.40 NAU3736b
78.99 JESPR63
85.30 Gh336
86.08 TMB0142
87.05 HAU0611
87.77 DPL0490
88.67 GLB2
89.96 TMB1869
92.36 BNL2921
93.76 JESPR240b
BNL2564a MGHES10
93.84 Gh216 NAU3533b
95.11 MUSS422a
96.79 CIR018
97.80 BNL1350a
98.55 Gh649a
98.93 NAU2182
98.94 JESPR56a
99.03 HAU0672b
100.69 NAU3365b
102.14 DPL0513
102.71 BNL2702
104.68 CIR049
106.12 TMB0062
107.70 BNL3090a
107.77 DPL0094
108.71 Gh350b
112.62 NAU4891
114.33 TMB2544
114.84 BNL3888
115.55 HAU1351
116.34 HAU0309
118.70 HAU1229
120.76 NAU4074
121.30 TMB0011
122.81 BNL3580
123.67 HAU0606
125.13 NAU4073b
127.59 CCRG087-SSCP
128.28 HAU0940c
128.95 HAU0940d
134.98 NAU5100a
147.63 NAU4044
148.66 NAU4045a
151.15 HAU2102
151.70 BNL2599
152.80 HAU2101
153.77 Gh75
156.65 HAU0282
158.20 HAU-SNP572
164.74 MGHES37
168.64 HAU1619a
170.58 HAU1166
177.78 BNL846
178.26 HAU-SNP504
179.64 HAU1045a
183.40 HAU2307b
186.87 CIR110b
0.00 MON_CGR5007
2.61 NAU3535
5.53 BNL2877
13.90 TMB1738
26.15 HAU-SNP432
38.87 CIR381
44.62 HAU-SNP329
46.38 BNL1434
51.73 BNL4060
56.76 BNL3513a
59.36 TMB2386a
62.32 CCRG028
64.38 HAU0296
65.04 BNL3547
65.71 HAU1980a
67.16 BNL3512
67.29 BNL1897
70.53 NAU5134
73.74 Gh669
74.46 NAU3189a
75.54 BNL2651
76.15 NAU3485a
77.26 MUSB0915
77.88 BNL3590
78.24 DPL0674
79.16 JESPR93
79.43 BNL1410
80.18 HAU-SNP323
80.44 CIR401b
81.58 DPL0046
81.95 HAU2154
81.96 JESPR101
82.71 HAU2643
83.57 HAU2917a
84.46 BNL3971
85.39 BNL3292
86.12 DPL0216
86.70 TMB0514
86.74 NAU3875
86.79 Gh198
87.08 NAU3626
88.15 HAU0306
88.55 MUSS073
89.56 HAU2923
93.43 NAU2908b
95.36 NAU4035b
98.16 HAU0880b
98.57 HAU2803
99.73 HAU-InDel014
100.84 NAU2761a
101.98 HAU1155
107.39 HAU2690
115.00 NAU3684
115.89 CIR376 JESPR304
123.45 HAU-SNP549
124.29 BNL663
126.47 HAU1798
133.14 NAU2858
140.16 HAU-SNP213
146.50 HAU2001
147.99 NAU3775
150.49 HAU2610
151.93 HAU2463
156.03 NAU3419c
0.00 MON_SHIN-1400
5.39 NAU5289
10.53 NAU2960a
11.02 CIR202
11.58 HAU0883a
12.41 CM129A
13.23 CIR228
13.84 JESPR107
15.06 CIR133
18.62 HAU2024
20.79 JESPR231
23.83 MGHES66
24.87 HAU1192a
31.87 HAU0875
32.99 HAU2127a
34.69 HAU1322
40.88 HAU-InDel203
41.33 BNL1080
48.80 NAU5035
50.12 HAU2511
50.95 HAU2588
52.85 BNL4017 MUSS162
53.00 HAU1221
57.21 NAU3573
57.44 NAU3541
57.69 DPL0605
59.20 BNL226 DPL0631
61.72 BNL244
63.22 DPL0733
64.44 NAU6371
65.25 BNL140
65.37 Gh129
65.67 MUSS172
66.04 BNL3398
69.27 HAU1468a
70.52 Gh681a
72.58 GhEXPA6-3-ds
75.00 HAU-SNP130
75.23 HAU0604
77.38 HAU-SNP092
78.14 MUSS444
78.37 NAU3382a
78.66 GhPEAMT-ds
79.05 NAU3309b
79.51 NAU3394
79.56 BNL3441
79.60 NAU2929 NAU2928
80.01 TMB2826
80.16 NAU3671
80.39 BNL3267
80.61 DPL0901b
81.30 HAU-SNP744
83.35 HAU0992
83.93 NAU5443b
85.39 HAU1624
87.90 HAU3176b
89.53 HAU2860b
90.16 BNL1379
93.19 TMB2069
94.59 HAU1420
100.39 HAU0920
103.81 JESPR303
104.38 CIR030
106.05 HAU-SNP737
111.24 BNL3408b
113.50 NAU5233
113.58 NAU3839b
114.34 NAU3479
127.95 GhTUB7-ss
137.95 NAU3172
139.99 HAU0292
142.43 MUCS547
156.23 NAU2836
0.00 HAU0751
11.26 HAU3302
13.86 NAU5236
20.05 HAU1895
21.12 HAU-InDel167
37.17 NAU3868c
41.18 MUSB1112b
47.52 HAU0101
52.71 HAU0036
53.56 DPL0451
54.82 HAU0086b
60.75 DPL0494
61.77 TMB2483b
62.65 HAU2016
62.97 JESPR223
63.98 NAU3093
68.04 Gh117
69.77 NAU2363
71.52 DPL0667
71.68 BNL530
72.46 HAU3371
72.94 HAU0980
73.71 BNL3988
74.02 NAU6672 NAU2654
75.96 MUSB1050a
76.68 HAU1300
82.69 Gh209
85.27 BNL2939
85.80 NAU3491
86.77 BNL3994
86.79 DPL0196
87.45 HAU1056a NAU5180b
87.75 HAU3120
88.15 DPL0273
88.86 NAU2672a
89.10 HAU1332
89.81 Gh124
90.20 HAU2568
90.71 BNL4047
91.11 DPL0299
91.29 HAU1588
91.36 NAU3938
93.87 HAU2085
94.72 NAU3825b
97.81 LMWHSP1-ds
99.06 GbIP20a
101.35 CIR222
105.85 Gh107
108.65 BNL2821b
114.16 DPL0573
129.33 HAU1400
134.30 CIR223
136.30 BNL3089
140.07 GbIDP56
0.00 TMB0478
9.05 HAU-InDel103
10.99 NAU3737
20.73 NAU3012a
23.87 CIR224
24.45 HAU2126
25.09 NAU3450 NAU1137
25.98 BNL2865
29.26 TMB1418
29.79 NAU3607
31.13 BNL1042
31.73 HAU0746
32.57 HAU1797
33.34 NAU3828
33.62 MUSS106
35.41 NAU4034
42.16 NAU3197b
43.51 HAU2311
45.55 NAU5387
60.26 CM183
62.64 BNL3020
63.76 JESPR310
65.74 HAU0633
66.91 DPL0838b
76.36 HAU0878a
78.37 NAU3416
80.81 DPL0225a
83.14 HAU2694
84.11 NAU5015
86.58 NAU5273
88.36 NAU1372b
90.40 NAU1372c
91.47 NAU5255a
93.27 HAU-SNP538
93.61 BNL3492b
95.99 BNL3029
96.09 NAU3650a
98.09 NAU3212
98.77 NAU2630
99.67 HAU1495
100.54 HAU2223
101.07 HAU2783a
101.43 DPL0368
102.20 NAU3935a
103.01 CIR373
104.71 HAU3372
106.15 Gh109a
106.66 NAU3097
107.68 HAU-SNP617
108.41 HAU1034
109.35 Gh381a
110.50 NAU5160
112.00 HAU1041
113.96 BNL2988
114.97 HAU1197
115.61 NAU3325
116.03 HAU1952b
116.05 HAU0535
116.37 DPL0156
116.88 BNL218
118.53 NAU2736
119.84 NAU2800a
120.94 HAU3292
121.34 HAU0627
122.90 TMB0193
123.98 HAU1269
127.44 HAU-InDel104
128.92 NAU2932b
129.74 NAU5299
133.79 MUCS108
134.13 NAU861
134.20 NAU3014
134.99 MUSS118
142.61 CIR401a
143.24 NAU3620
145.11 HAU5088
146.39 JESPR197
146.53 NAU3001b
146.77 NAU5417
146.97 BNL3992
149.25 Gh83
149.66 BNL2448
150.88 HAU1500
151.21 HAU0032
152.20 HAU2313
156.71 HAU1168
157.90 HAU1385
159.62 HAU1384
163.64 HAU1976a
164.21 HAU0506
168.51 NAU2140
172.17 Gh594
174.51 HAU3183
175.15 NAU4031
176.32 Gh211
177.12 HAU1404
177.66 Gh422
180.88 HAU2621
181.09 TMB2897b
181.54 MUSB0977b
181.70 CIR301
181.86 NAU3569
183.18 CIR294
184.29 GhDET3-ds
184.64 NAU5099b
184.82 NAU5046a
185.56 DPL0810
186.25 HAU2536
186.43 BNL3955
187.10 DPL0637
187.54 Gh166a
187.99 NAU4898
188.98 NAU5149
190.09 NAU2562
192.61 HAU-SNP123
193.33 Gh262
196.27 DPL0837
196.98 NAU2561
198.01 NAU3402
198.42 HAU0968
201.18 NAU3382b
201.72 BNL2732
201.95 CM65 CIR185
203.02 JESPR50
203.90 NAU5400
204.53 BNL1038
206.70 HAU-InDel140
208.21 HAU0042a
210.36 Gh206
212.18 CIR253
215.01 HAU2691a
221.99 NAU4057
228.57 NAU3036
231.99 HAU1496
235.24 MUSS219
237.73 HAU3401a
242.76 HAU0576
0.00 STV106
7.79 NAU2714b
10.35 NAU2713b
10.58 JESPR119
14.61 DPL0328
16.41 BNL1902
17.52 BNL584
22.12 HAU1481
28.03 Gh39
30.03 DPL0588
32.78 TMB0154
34.46 Gh32
38.41 DPL0035
40.63 NAU5270a
41.90 BNL1746
45.54 NAU4969b
49.62 HAU0975
50.86 NAU3243a
52.41 NAU5269
52.86 DPL0238
53.13 BNL4004
55.96 DPL0101
56.70 CIR280
59.05 TMB1530
62.13 Gh100b
63.05 JESPR273
68.21 NAU2156
69.17 Gh82b
70.55 DPL0847
71.99 DPL0080
73.83 DPL0681
74.77 HAU0376
77.09 BNL1112
78.46 DPL0153
80.86 CS097
86.31 HAU2467a
86.91 CIR233
88.68 BNL3295
90.80 JESPR247
91.47 BNL861
91.72 BNL3812
92.75 HAU2768
93.18 HAU2119
93.79 HAU3552
94.89 TMB2958
95.03 HAU1293
95.82 BNL1064
96.62 HAU0668
96.85 HAU-SNP232
96.99 TMB2959
97.02 MUSB1064
97.03 MUSB1164
97.05 HAU1488 HAU1537
NAU2679d
97.11 HAU0091
101.32 HAU0483
101.35 NAU5434 NAU5433
101.67 GhFb-B6-2-ds
102.58 HAU0196
103.88 JESPR194
104.21 HAU0210
105.10 NAU3803
105.94 NAU2968
108.03 BNL3650
109.95 NAU4946
112.86 NAU3365a
115.61 BNL2884
118.35 DPL0665a
119.52 HAU0283a
124.71 TMB1922
127.43 GhCFE
129.95 HAU1143
132.25 Gh185
138.09 NAU3206
148.50 HAU0504
149.72 NAU3677b
152.86 BNL2823
154.27 CIR128
155.71 HAU1056b
159.72 NAU2773a
168.10 Gh513
171.43 NAU3427

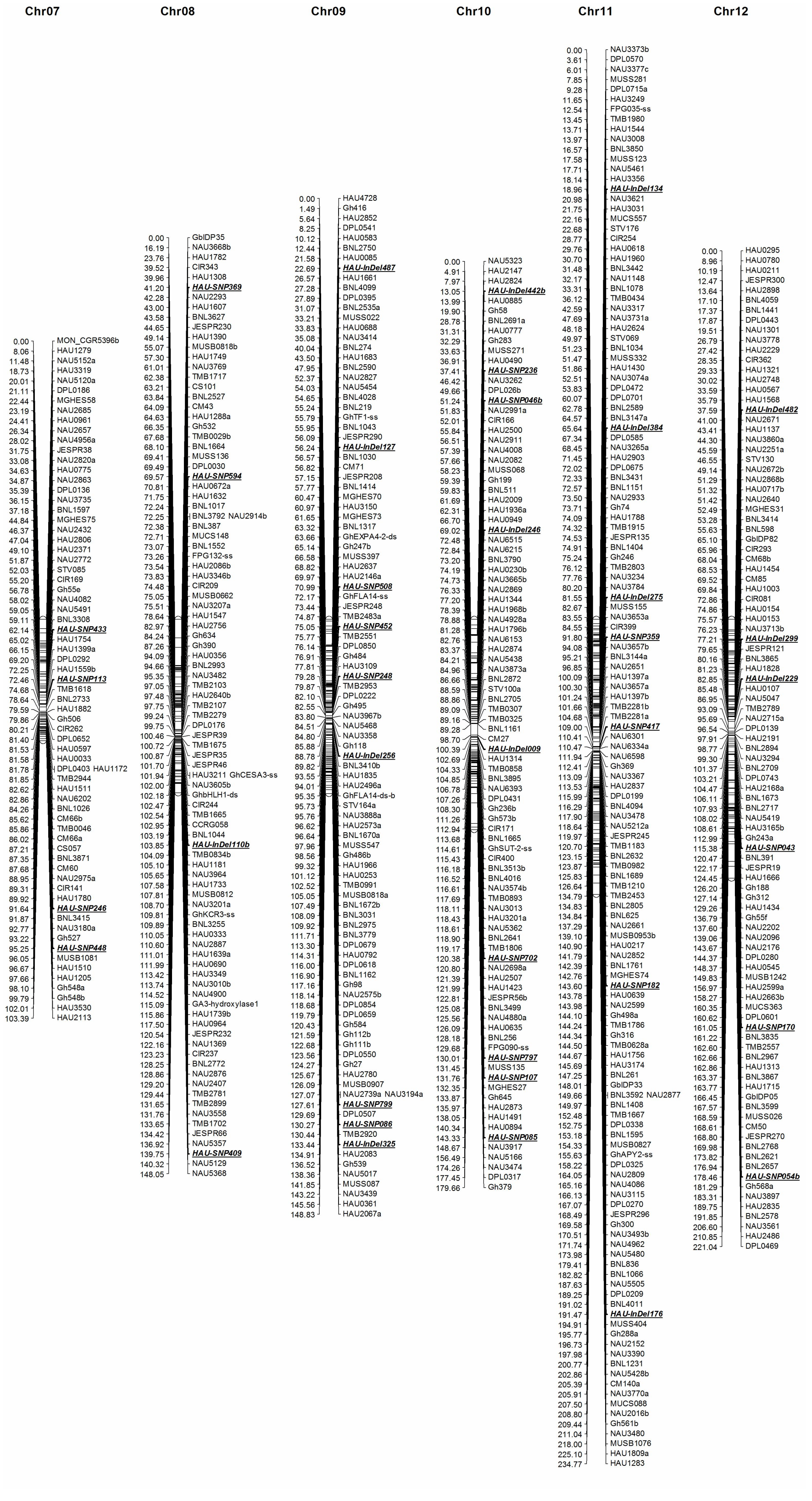
Chr07
0.00 MON_CGR5396b
8.06 HAU1279
11.48 NAU5152a
18.73 HAU3319
20.01 NAU5120a
21.11 DPL0186
22.44 MGHES58
23.19 NAU2685
24.41 HAU0961
26.34 NAU2657
28.02 NAU4956a
31.75 JESPR38
33.08 NAU2820a
34.63 HAU0775
34.87 NAU2863
35.39 DPL0136
36.15 NAU3735
37.18 BNL1597
44.84 MGHES75
46.37 NAU2432
47.04 HAU2806
49.10 HAU2371
51.87 NAU2772
52.03 STV085
55.20 CIR169
56.78 Gh55e
58.02 NAU4082
59.05 NAU5491
59.11 BNL3308
62.14 HAU-SNP433
65.02 HAU1754
66.15 HAU1399a
69.20 DPL0292
72.25 HAU1559b
72.46 HAU-SNP113
74.68 TMB1618
78.64 BNL2733
79.59 HAU1882
79.86 Gh506
80.21 CIR262
81.40 DPL0652
81.53 HAU0597
81.58 HAU0033
81.78 DPL0403 HAU1172
81.85 TMB2944
82.62 HAU1511
82.86 NAU6202
84.26 BNL1026
85.62 CM66b
85.86 TMB0046
86.02 CM66a
87.21 CS057
87.35 BNL3871
87.68 CM60
88.95 NAU2975a
89.31 CIR141
89.92 HAU1780
91.64 HAU-SNP246
91.87 BNL3415
92.77 NAU3180a
93.22 Gh527
95.25 HAU-SNP448
96.05 MUSB1081
96.67 HAU1510
97.66 HAU1205
98.10 Gh548a
99.79 Gh548b
102.01 HAU3530
103.39 HAU2113
Chr08
0.00 GbIDP35
16.19 NAU3668b
23.76 HAU1782
39.52 CIR343
39.96 HAU1308
41.20 HAU-SNP369
42.28 NAU2293
43.00 HAU1607
43.58 BNL3627
44.65 JESPR230
49.14 HAU1390
55.07 MUSB0818b
57.30 HAU1749
61.01 NAU3769
62.38 TMB1717
63.21 CS101
63.84 BNL2527
64.09 CM43
64.63 HAU1288a
66.35 Gh532
67.68 TMB0029b
68.10 BNL1664
69.41 MUSS136
69.49 DPL0030
69.57 HAU-SNP594
70.81 HAU0672a
71.75 HAU1632
72.24 BNL1017
72.25 BNL3792 NAU2914b
72.38 BNL387
72.71 MUCS148
73.07 BNL1552
73.26 FPG132-ss
73.54 HAU2086b
73.83 HAU3346b
74.48 CIR209
75.05 MUSB0662
75.51 NAU3207a
78.64 HAU1547
82.97 HAU2756
84.24 Gh634
87.26 Gh390
94.09 HAU0356
94.66 BNL2993
95.35 NAU3482
97.05 TMB2103
97.48 HAU2640b
97.75 TMB2107
99.24 TMB2279
99.75 DPL0176
100.46 JESPR39
100.72 TMB1675
100.87 JESPR35
101.70 JESPR46
101.94 HAU3211 GhCESA3-ss
102.00 NAU3605b
102.18 GhbHLH1-ds
102.47 CIR244
102.54 TMB1665
102.95 CCRG058
103.19 BNL1044
103.85 HAU-InDel110b
104.09 TMB0834b
105.10 HAU1181
105.65 NAU3964
107.58 HAU1733
107.81 MUSB0812
108.70 NAU3201a
109.81 GhKCR3-ss
109.89 BNL3255
110.05 HAU0333
110.60 NAU2887
111.01 HAU1639a
111.99 HAU0690
113.42 HAU3349
113.74 NAU3010b
114.52 NAU4900
115.09 GA3-hydroxylase1
115.86 HAU1739b
117.50 HAU0964
120.54 JESPR232
122.16 NAU1369
123.23 CIR237
128.25 BNL2772
128.86 NAU2876
129.20 NAU2407
129.44 TMB2781
131.65 TMB2899
131.76 NAU3558
133.65 TMB1702
134.42 JESPR66
136.92 NAU5357
139.75 HAU-SNP409
140.32 NAU5129
148.05 NAU5368
Chr09
0.00 HAU4728
1.49 Gh416
5.64 HAU2852
8.25 DPL0541
10.12 HAU0583
12.44 BNL2750
21.58 HAU0085
22.69 HAU-InDel487
26.57 HAU1661
27.28 BNL4099
27.89 DPL0395
31.07 BNL2535a
33.21 MUSS022
33.83 HAU0688
35.08 NAU3414
40.04 BNL274
43.50 HAU1683
47.95 BNL2590
52.37 NAU2827
54.03 NAU5454
54.65 NAU4028
55.24 BNL219
55.79 GhTF1-ss
55.95 BNL1043
56.09 JESPR290
56.24 HAU-InDel127
56.57 BNL1030
56.82 CM71
57.15 JESPR208
57.77 BNL1414
60.47 MGHES70
60.97 HAU3150
61.65 MGHES73
63.32 BNL1317
63.66 GhEXPA4-2-ds
65.14 Gh247b
66.58 MUSS397
68.82 HAU2637
69.97 HAU2146a
70.99 HAU-SNP508
72.17 GhFLA14-ss
73.44 JESPR248
74.87 TMB2483a
75.05 HAU-SNP452
75.77 TMB2551
76.14 DPL0850
76.91 Gh484
77.81 HAU3109
79.28 HAU-SNP248
79.87 TMB2953
82.10 DPL0222
82.55 Gh495
83.80 NAU3967b
84.51 NAU5468
84.80 NAU3358
85.88 Gh118
88.78 HAU-InDel256
89.82 BNL3410b
93.55 HAU1835
94.01 HAU2496a
95.35 GhFLA14-ds-b
95.73 STV164a
95.76 NAU3888a
96.62 HAU2573a
96.64 BNL1670a
97.96 MUSS547
98.56 Gh486b
99.32 HAU1966
101.12 HAU0253
102.52 TMB0991
105.05 MUSB0818a
107.49 BNL1672b
108.09 BNL3031
109.92 BNL2975
111.71 BNL3779
113.30 DPL0679
114.31 HAU0792
116.00 DPL0618
116.90 BNL1162
117.16 Gh98
118.14 NAU2575b
118.68 DPL0854
119.79 DPL0659
120.43 Gh584
121.59 Gh112b
122.68 Gh111b
123.56 DPL0550
124.27 Gh27
125.67 HAU2780
126.09 MUSB0907
127.07 NAU2739a NAU3194a
127.61 HAU-SNP799
129.69 DPL0507
130.27 HAU-SNP086
130.44 TMB2920
133.44 HAU-InDel325
134.91 HAU2083
136.52 Gh539
138.36 NAU5017
141.85 MUSS087
143.22 NAU3439
145.56 HAU0361
148.83 HAU2067a
Chr10
0.00 NAU5323
4.91 HAU2147
7.97 HAU2824
13.05 HAU-InDel442b
13.99 HAU0885
19.90 Gh58
28.78 BNL2691a
31.31 HAU0777
32.29 Gh283
33.63 MUSS271
36.91 HAU0490
37.41 HAU-SNP236
46.42 NAU3262
49.66 DPL026b
51.24 HAU-SNP046b
51.83 NAU2991a
52.01 CIR166
55.84 HAU2500
56.51 NAU2911
57.39 NAU4008
57.66 NAU2082
58.23 MUSS068
59.39 Gh199
59.83 BNL511
61.69 HAU2009
62.31 HAU1936a
66.70 HAU0949
69.02 HAU-InDel246
72.48 NAU6155
72.84 NAU6215
73.20 BNL3790
74.19 HAU0230b
74.73 NAU3665b
76.33 NAU2869
77.20 HAU1344
78.39 HAU1968b
78.88 NAU4928a
81.28 HAU1796b
82.76 NAU6153
83.37 HAU2874
84.21 NAU5438
84.96 NAU3873a
86.66 BNL2872
88.59 STV100a
88.86 BNL2705
89.09 TMB0307
89.16 TMB0325
89.28 BNL1161
98.70 CM27
100.39 HAU-InDel009
102.69 HAU1314
104.33 TMB0858
104.85 BNL3895
106.78 NAU6393
107.26 DPL0431
108.30 Gh236b
111.26 Gh573b
112.94 CIR171
113.68 BNL1665
114.61 GhSUT-2-ss
115.43 CIR400
116.18 BNL3513b
116.52 BNL4016
116.61 NAU3574b
117.69 TMB0893
118.11 NAU3013
118.43 HAU3201a
118.61 NAU5362
118.90 BNL2641
119.17 TMB1806
120.38 HAU-SNP702
120.80 NAU2698a
121.39 HAU2507
121.99 HAU1423
122.81 JESPR56b
125.08 BNL3499
125.56 NAU4880a
126.09 HAU0635
128.18 BNL256
129.68 FPG090-ss
130.01 HAU-SNP797
131.45 MUSS135
131.76 HAU-SNP107
132.35 MGHES27
133.87 Gh645
135.97 HAU2873
138.05 HAU1491
140.34 HAU0894
143.33 HAU-SNP085
148.67 NAU3917
156.49 NAU5166
174.26 NAU3474
177.45 DPL0317
179.66 Gh379
Chr11
0.00 NAU3373b
3.61 DPL0570
6.01 NAU3377c
7.85 MUSS281
9.28 DPL0715a
11.65 HAU3249
12.54 FPG035-ss
13.45 TMB1980
13.71 HAU1544
13.97 NAU3008
16.57 BNL3850
17.58 MUSS123
17.71 NAU5461
18.14 HAU3356
18.96 HAU-InDel134
20.98 NAU3621
21.75 HAU3031
22.16 MUCS557
22.68 STV176
28.77 CIR254
29.76 HAU0618
30.70 HAU1960
31.48 BNL3442
32.17 NAU1148
33.31 BNL1078
36.12 TMB0434
42.59 NAU3317
47.69 NAU3731a
48.18 HAU2624
49.97 STV069
51.23 BNL1034
51.47 MUSS332
51.86 HAU1430
52.51 NAU3074a
53.83 DPL0472
60.07 DPL0701
62.78 BNL2589
64.57 BNL3147a
65.64 HAU-InDel384
67.34 DPL0585
68.45 NAU3265a
71.45 HAU2903
72.02 DPL0675
72.33 BNL3431
72.57 BNL1151
73.50 NAU2933
73.71 Gh74
74.09 HAU1788
74.32 TMB1915
74.53 JESPR135
74.91 BNL1404
75.24 Gh246
76.12 TMB2803
77.76 NAU3234
80.20 NAU3784
81.55 HAU-InDel275
82.67 MUSS155
83.55 NAU3653a
84.55 CIR399
91.80 HAU-SNP359
94.08 NAU3657b
95.21 BNL3144a
96.85 NAU2651
100.09 HAU1397a
100.30 NAU3657a
101.24 HAU1397b
101.66 TMB2281b
104.68 TMB2281a
109.00 HAU-SNP417
110.41 NAU6301
110.47 NAU6334a
111.94 NAU6598
112.41 Gh369
113.09 NAU3367
113.53 HAU2837
115.99 DPL0199
116.29 BNL4094
117.90 NAU3478
118.64 NAU5212a
119.97 JESPR245
120.70 TMB1183
123.15 BNL2632
123.87 TMB0982
125.83 BNL1689
126.64 TMB1210
134.79 TMB2453
134.83 BNL2805
134.84 BNL625
137.29 NAU2661
139.10 MUSB0953b
140.90 HAU0217
141.79 NAU2852
142.39 BNL1761
142.76 MGHES74
143.60 HAU-SNP182
143.78 HAU0639
143.98 NAU2599
144.10 Gh498a
144.24 TMB1786
144.34 Gh316
144.50 TMB0628a
144.67 HAU1756
145.69 HAU3174
147.25 BNL261
148.01 GbIDP33
149.66 BNL3592 NAU2877
149.97 BNL1408
152.48 TMB1667
152.75 DPL0338
153.18 BNL1595
154.33 MUSB0827
155.63 GhAPY2-ss
158.22 DPL0325
164.05 NAU2809
165.16 NAU4086
166.13 NAU3115
167.07 DPL0270
168.49 JESPR296
169.58 Gh300
170.51 NAU3493b
171.74 NAU4962
173.98 NAU5480
179.41 BNL836
182.82 BNL1066
187.63 NAU5505
189.25 DPL0209
191.02 BNL4011
191.47 HAU-InDel176
194.91 MUSS404
195.77 Gh288a
196.73 NAU2152
197.98 NAU3390
200.77 BNL1231
202.86 NAU5428b
205.39 CM140a
205.91 NAU3770a
207.50 MUCS088
208.80 NAU2016b
209.44 Gh561b
211.04 NAU3480
218.00 MUSB1076
225.10 HAU1809a
234.77 HAU1283
Chr12
0.00 HAU0295
8.96 HAU0780
10.19 HAU0211
12.47 JESPR300
13.64 HAU2898
17.10 BNL4059
17.37 BNL1441
17.87 DPL0443
19.51 NAU1301
26.79 NAU3778
27.42 HAU2229
28.35 CIR362
29.33 HAU1321
30.02 HAU2748
33.59 HAU0567
35.79 HAU1568
37.59 HAU-InDel482
41.00 NAU2671
43.41 HAU1137
44.30 NAU3860a
45.59 NAU2251a
46.55 STV130
49.14 NAU2672b
51.29 NAU2868b
51.32 HAU0717b
51.42 NAU2640
52.49 MGHES31
53.28 BNL3414
55.63 BNL598
65.10 GbIDP82
65.96 CIR293
68.04 CM68b
68.53 HAU1454
69.52 CM85
69.84 HAU1003
72.86 CIR081
74.86 HAU0154
75.57 HAU0153
76.23 NAU3713b
77.21 HAU-InDel299
79.65 JESPR121
80.16 BNL3865
81.23 HAU1828
82.85 HAU-InDel229
85.48 HAU0107
86.95 NAU5047
93.09 TMB2789
95.69 NAU2715a
96.54 DPL0139
97.91 HAU2191
98.77 BNL2894
99.30 NAU3294
101.37 BNL2709
103.21 DPL0743
104.47 HAU2168a
106.11 BNL1673
107.93 BNL2717
108.02 NAU5419
108.61 HAU3165b
112.99 Gh243a
115.38 HAU-SNP043
120.47 BNL391
122.17 JESPR19
124.45 HAU1666
126.20 Gh188
127.14 Gh312
129.26 HAU1434
136.79 Gh55f
137.60 NAU2202
139.06 NAU2096
143.67 NAU2176
144.37 DPL0280
148.37 HAU0545
149.83 MUSB1242
156.97 HAU2599a
158.27 HAU2663b
160.35 MUCS363
160.62 DPL0601
161.05 HAU-SNP170
161.22 BNL3835
162.60 TMB2557
162.66 BNL2967
162.86 HAU1313
163.37 BNL3867
163.77 HAU1715
166.45 GbIDP05
167.57 BNL3599
168.59 MUSS026
168.61 CM50
168.80 JESPR270
169.98 BNL2768
173.82 BNL2621
176.94 BNL2657
178.46 HAU-SNP054b
181.29 Gh568a
183.31 NAU3897
189.75 HAU2835
191.85 BNL2578
206.60 NAU3561
210.85 HAU2486
221.04 DPL0469

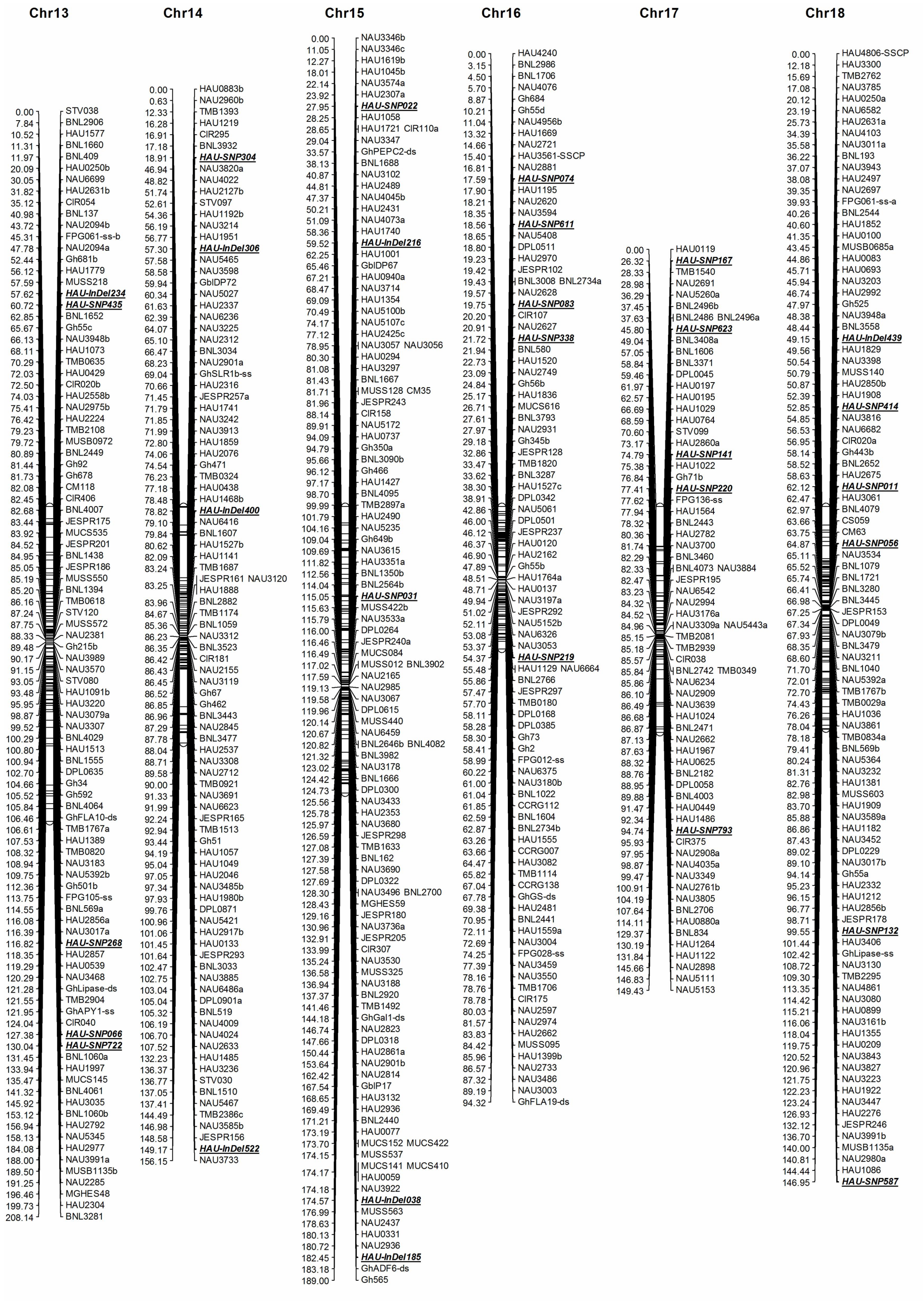

Chr13
Chr14
Chr15
Chr16
Chr17
Chr18

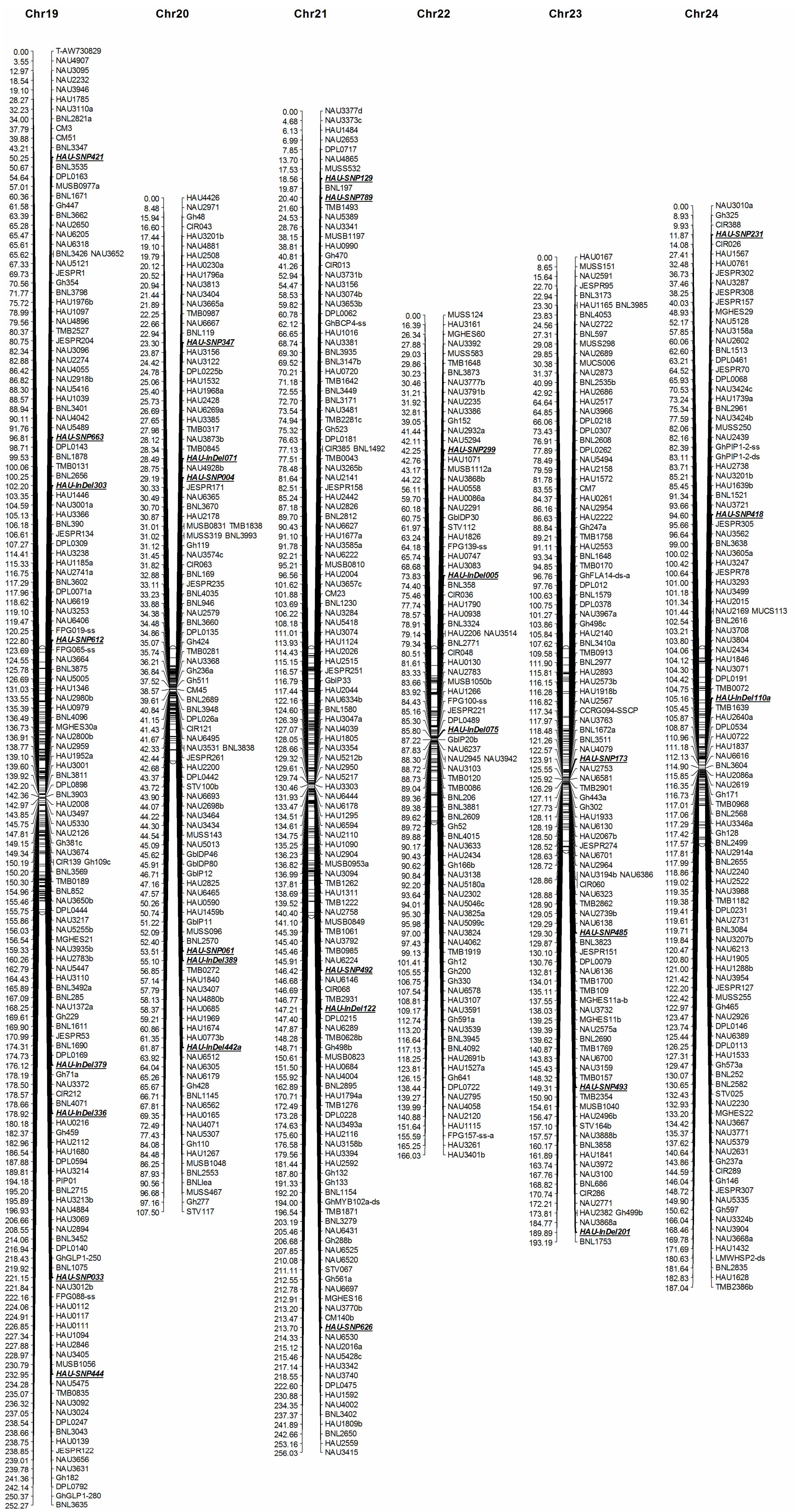
Chr19
0.00
3.55
12.97
18.54
19.10
28.27
32.23
34.00
37.79
39.88
43.21
50.25
50.67
54.64
57.01
60.36
61.58
63.39
65.28
65.47
65.61
65.62
67.33
69.73
70.56
71.77
75.72
78.99
79.56
80.37
80.75
82.34
82.88
86.42
86.82
88.30
88.57
88.94
90.11
91.76
96.81
98.71
99.53
100.06
100.25
102.20
103.35
104.59
105.13
106.18
106.61
107.27
114.41
115.33
116.75
117.29
117.96
118.62
119.10
119.47
120.25
122.80
123.69
124.55
125.78
126.69
131.03
133.55
135.39
136.49
136.73
136.91
138.77
139.10
139.60
139.92
142.20
142.36
142.97
143.85
145.75
147.81
149.15
149.34
150.19
150.20
150.30
154.96
155.46
155.75
155.86
156.03
156.54
159.33
160.26
162.79
164.43
165.89
167.09
168.25
169.61
169.90
170.99
174.31
174.73
176.12
178.19
178.50
178.57
178.66
179.82
180.18
182.37
182.96
186.54
187.88
189.81
194.18
195.20
195.89
196.93
206.66
208.55
214.06
216.94
218.43
219.92
221.15
221.84
222.16
224.06
224.91
226.85
227.34
227.88
228.97
230.79
232.95
234.28
235.07
236.32
237.05
238.54
238.66
238.75
238.85
239.01
239.78
241.36
242.14
250.37
252.27
T-AW730829
NAU4907
NAU3095
NAU2232
NAU3946
HAU1785
NAU3110a
BNL2821a
CM3
CM51
BNL3347
HAU-SNP421
BNL3535
DPL0163
MUSB0977a
BNL1671
Gh447
BNL3662
NAU2650
NAU6205
NAU6318
BNL3426 NAU3652
NAU5121
JESPR1
Gh354
BNL3798
HAU1976b
HAU1097
NAU4896
TMB2527
JESPR204
NAU3096
NAU2274
NAU4055
NAU2918b
NAU5416
HAU1039
BNL3401
NAU4042
NAU5489
HAU-SNP663
DPL0143
BNL1878
TMB0131
BNL2656
HAU-InDel303
HAU1446
NAU3001a
HAU3366
BNL390
JESPR134
DPL0309
HAU3238
HAU1185a
NAU2741a
BNL3602
DPL0071a
NAU6619
NAU3253
NAU6406
FPG019-ss
HAU-SNP612
FPG065-ss
NAU3664
BNL3875
NAU5005
HAU1346
NAU2980b
HAU0979
BNL4096
MGHES30a
NAU2800b
NAU2959
HAU1952a
HAU3001
BNL3811
DPL0898
BNL3903
HAU2008
HAU3497
NAU5330
NAU2126
Gh381c
NAU3674
CIR139 Gh109c
BNL3569
TMB0189
BNL852
NAU3650b
DPL0444
NAU3217
NAU5255b
MGHES21
NAU3935b
HAU2783b
NAU5447
HAU3110
BNL3492a
BNL285
NAU1372a
Gh229
BNL1611
JESPR53
BNL1690
DPL0169
HAU-InDel379
Gh71a
NAU3372
CIR212
BNL4071
HAU-InDel336
HAU0216
Gh459
HAU2112
HAU1680
DPL0594
HAU3214
PIP01
BNL2715
HAU3213b
NAU4884
HAU3069
NAU2894
BNL3452
DPL0140
GhGLP1-250
BNL1075
HAU-SNP033
NAU3012b
FPG088-ss
HAU0112
HAU0117
HAU0111
HAU1094
HAU2846
NAU3405
MUSB1056
HAU-SNP444
NAU5475
TMB0835
NAU3092
NAU3024
DPL0247
BNL3043
HAU0139
JESPR122
NAU3656
NAU3631
Gh182
DPL0792
GhGLP1-280
BNL3635
Chr20
0.00
8.48
15.94
16.60
17.44
19.10
19.79
20.12
20.52
20.94
21.44
21.89
22.25
22.66
22.94
23.30
23.87
24.42
24.78
25.06
25.40
25.73
26.69
27.65
27.98
28.12
28.34
28.49
28.75
29.19
30.33
30.49
30.70
30.87
31.01
31.02
31.12
31.45
31.82
32.88
33.11
33.23
33.88
34.38
34.48
34.86
35.07
35.74
36.21
36.84
37.52
38.57
39.61
40.84
41.15
41.43
41.67
42.33
42.44
42.68
43.37
43.72
43.90
44.07
44.22
44.30
44.54
45.09
45.62
45.91
46.71
47.16
47.57
50.26
50.74
51.22
52.09
52.40
53.51
55.10
56.85
57.14
57.79
58.13
58.37
59.21
60.86
61.35
61.87
63.92
64.04
65.26
65.67
66.71
67.81
69.35
72.49
77.43
84.08
84.48
86.25
87.93
90.56
96.68
97.16
107.50
HAU4426
NAU2971
Gh48
CIR043
HAU3201b
NAU4881
HAU2508
HAU0230a
HAU1796a
NAU3813
NAU3404
NAU3665a
TMB0987
NAU6667
BNL119
HAU-SNP347
HAU3156
NAU3122
DPL0225b
HAU1532
HAU1968a
HAU2428
NAU6269a
HAU3385
TMB0317
NAU3873b
TMB0845
HAU-InDel071
NAU4928b
HAU-SNP004
JESPR171
NAU6365
BNL3670
HAU2178
MUSB0831 TMB1838
MUSS319 BNL3993
Gh119
NAU3574c
CIR063
BNL169
JESPR235
BNL4035
BNL946
NAU2579
BNL3660
DPL0135
Gh424
TMB0281
NAU3368
Gh236a
Gh511
CM45
BNL2689
BNL3948
DPL026a
CIR121
NAU6495
NAU3531 BNL3838
JESPR261
HAU2200
DPL0442
STV100b
NAU6693
NAU2698b
NAU3464
NAU3434
MUSS143
NAU5013
GbIDP46
GbIDP80
GbIP12
HAU2825
NAU6465
HAU0590
HAU1459b
GbIP11
MUSS096
BNL2570
HAU-SNP061
HAU-InDel389
TMB0272
HAU1840
NAU3407
NAU4880b
HAU0685
HAU1969
HAU1674
HAU0773b
HAU-InDel442a
NAU6512
NAU6305
NAU6179
Gh428
BNL1145
NAU6562
HAU0165
NAU4071
NAU5307
Gh110
HAU1267
MUSB1048
BNL2553
BNLlea
MUSS467
Gh277
STV117
Chr21
0.00
4.68
6.13
6.99
7.85
13.70
17.53
18.56
19.87
20.40
21.60
24.53
28.76
38.15
38.81
40.81
41.26
52.94
54.47
58.53
59.82
60.78
62.12
66.65
68.74
69.30
69.52
70.21
71.18
72.55
72.70
73.54
74.94
75.32
76.63
77.13
77.51
78.48
81.64
82.51
85.24
87.18
89.70
90.43
91.10
91.78
92.21
95.21
96.56
101.62
101.88
103.69
106.22
108.18
111.01
113.93
114.43
115.15
116.57
116.79
117.44
122.16
124.60
126.39
127.07
128.05
128.66
129.32
129.61
129.74
130.46
131.93
133.47
134.51
134.61
134.75
135.25
136.23
136.82
136.99
137.81
138.69
139.52
140.40
141.10
145.39
145.40
145.46
145.91
146.42
146.68
146.69
146.77
147.21
147.40
147.87
148.28
148.71
150.61
151.50
155.92
162.89
170.71
172.49
173.28
174.63
175.60
176.58
179.56
181.44
187.80
191.33
192.20
194.00
196.54
203.19
205.46
206.68
207.85
210.08
211.11
212.55
212.78
212.91
213.20
213.47
213.70
214.33
215.12
215.46
217.14
218.55
222.60
230.88
234.35
237.37
241.89
242.66
253.16
256.03
NAU3377d
NAU3373c
HAU1484
NAU2653
DPL0717
NAU4865
MUSS532
HAU-SNP129
BNL197
HAU-SNP789
TMB1493
NAU5389
NAU3341
MUSB1197
HAU0990
Gh470
CIR013
NAU3731b
NAU3156
NAU3074b
NAU3653b
DPL0062
GhBCP4-ss
HAU1016
NAU3381
BNL3935
BNL3147b
HAU0720
TMB1642
BNL3449
BNL3171
NAU3481
TMB2281c
Gh523
DPL0181
CIR385 BNL1492
TMB0043
NAU3265b
NAU2141
JESPR158
HAU2442
NAU2826
BNL2812
NAU6627
HAU1677a
NAU3585a
NAU6222
MUSB0810
HAU2004
NAU3657c
CM23
BNL1230
NAU3284
NAU5418
HAU3074
HAU1124
HAU2026
HAU2515
JESPR251
GbIP33
HAU2044
NAU6334b
BNL1580
HAU3047a
NAU4039
HAU1805
NAU3354
NAU5212b
NAU2950
NAU5217
HAU3303
NAU6444
NAU6178
HAU1295
NAU6594
NAU2110
HAU1090
NAU2904
MUSB0953a
NAU3094
TMB1262
HAU1311
TMB1222
NAU2758
MUSB0849
TMB1061
NAU3792
TMB0985
NAU6224
HAU-SNP492
NAU6146
CIR068
TMB2931
HAU-InDel122
DPL0215
NAU6289
TMB0628b
Gh498b
MUSB0823
HAU0684
NAU4004
BNL2895
HAU1794a
TMB1276
DPL0228
NAU3493a
HAU2116
NAU3158b
HAU3394
HAU2592
Gh132
Gh133
BNL1154
GhMYB102a-ds
TMB1871
BNL3279
NAU6431
Gh288b
NAU6525
NAU6520
STV067
Gh561a
NAU6697
MGHES16
NAU3770b
CM140b
HAU-SNP626
NAU6530
NAU2016a
NAU5428c
HAU3342
NAU3740
DPL0475
HAU1592
NAU4002
BNL3402
HAU1809b
BNL2650
HAU2559
NAU3415
Chr22
0.00
16.39
26.34
27.88
29.03
29.86
30.23
30.46
31.21
31.92
32.81
39.05
41.44
42.11
42.25
42.76
43.17
44.22
56.11
59.70
60.18
60.75
61.97
63.24
64.18
65.74
68.68
73.83
74.40
75.46
77.74
78.57
78.91
79.14
79.34
80.51
81.61
83.33
83.66
83.92
84.43
85.16
85.30
85.80
87.22
87.83
88.30
88.72
88.73
89.04
89.36
89.38
89.62
89.72
89.88
90.17
90.43
90.62
90.84
92.20
93.64
94.01
95.30
95.98
97.00
97.43
99.13
101.41
105.55
106.75
107.54
108.81
109.17
112.74
113.20
116.64
117.13
118.25
123.81
126.15
138.44
139.27
139.99
140.88
151.64
155.59
165.25
166.03
MUSS124
HAU3161
MGHES60
NAU3392
MUSS583
TMB1648
BNL3873
NAU3777b
NAU3791b
NAU2235
NAU3386
Gh152
NAU2932a
NAU5294
HAU-SNP299
HAU1071
MUSB1112a
NAU3868b
HAU0558
HAU0086a
NAU2291
GbIDP30
STV112
HAU1826
FPG139-ss
HAU0747
HAU3083
HAU-InDel005
BNL358
CIR036
HAU1790
HAU0938
BNL3324
HAU2206 NAU3514
BNL2771
CIR048
HAU0130
NAU2783
MUSB1050b
HAU1266
FPG100-ss
JESPR221
DPL0489
HAU-InDel075
GbIP20b
NAU6237
NAU2945 NAU3942
NAU3103
TMB0120
TMB0086
BNL206
BNL3881
BNL2609
Gh52
BNL4015
NAU3633
HAU2434
Gh166b
NAU3138
NAU5180a
NAU2302
NAU5046c
NAU3825a
NAU5099c
NAU3824
NAU4062
TMB1919
Gh12
Gh200
Gh330
NAU6578
HAU3107
NAU3591
Gh591a
NAU3539
BNL3945
BNL4092
HAU2691b
HAU1527a
Gh641
DPL0722
NAU2795
NAU4058
NAU2120
HAU1115
FPG157-ss-a
HAU3261
HAU3401b
Chr23
0.00
8.65
15.64
22.70
22.94
23.30
23.83
24.56
27.31
29.08
29.85
30.38
31.37
40.99
42.92
64.64
64.85
66.06
73.43
76.91
77.89
78.49
79.59
81.78
83.55
84.37
86.16
86.63
88.84
89.21
91.11
93.34
94.85
96.76
97.76
100.63
100.75
101.27
103.86
105.84
107.62
109.58
111.90
115.81
116.15
116.28
116.82
117.34
117.97
118.48
121.26
122.57
123.91
125.55
125.92
126.29
127.11
127.73
128.11
128.19
128.50
128.52
128.63
128.72
128.86
128.88
128.90
129.05
129.29
129.30
129.87
130.10
130.76
132.81
134.01
135.11
137.55
138.03
139.25
139.62
140.87
143.83
145.43
148.32
149.31
150.90
154.61
156.47
157.10
160.17
161.89
163.74
167.76
168.82
170.74
172.21
173.81
184.77
189.89
193.19
HAU0167
MUSS151
NAU2591
JESPR95
BNL3173
HAU1165 BNL3985
BNL4053
NAU2722
BNL597
MUSS298
NAU2689
MUCS006
NAU2873
BNL2535b
HAU2686
HAU2517
NAU3966
DPL0218
DPL0307
BNL2608
DPL0262
NAU5494
NAU2158
HAU1572
CM7
HAU0261
NAU2954
HAU2222
Gh247a
TMB1758
HAU2553
BNL1648
TMB0170
GhFLA14-ds-a
DPL012
BNL1579
DPL0378
NAU3967a
Gh498c
HAU2140
BNL3410a
TMB0913
BNL2977
HAU2893
HAU2573b
HAU1918b
NAU2567
CCRG094-SSCP
NAU3763
BNL1672a
BNL3511
NAU4079
HAU-SNP173
NAU2753
NAU6581
TMB2901
Gh443a
Gh302
HAU1933
NAU6130
HAU2067b
JESPR274
NAU6701
NAU2964
NAU3194b NAU6386
CIR060
NAU6323
TMB2862
NAU2739b
HAU-SNP485
BNL3823
JESPR151
DPL0079
NAU6136
TMB1700
TMB109
MGHES11a-b
NAU3732
MGHES11b
NAU2575a
BNL2690
TMB1769
NAU6700
NAU3159
TMB0157
HAU-SNP493
TMB2354
MUSB1040
HAU2496b
STV164b
NAU3888b
BNL3858
HAU1841
NAU3972
NAU3100
BNL686
CIR286
NAU2771
NAU2382 Gh499b
NAU3868a
HAU-InDel201
BNL1753
Chr24
0.00
8.93
9.93
11.87
14.08
27.41
32.48
36.73
37.46
38.25
40.03
48.93
52.17
57.85
60.06
62.60
63.21
64.52
65.93
70.53
73.24
75.34
77.59
82.06
82.16
82.39
83.11
83.71
85.21
85.45
91.34
93.66
94.60
95.66
96.64
99.00
100.02
100.42
100.64
101.00
101.18
101.34
101.44
102.54
103.21
103.80
104.06
104.12
104.30
104.42
104.75
105.16
105.45
105.87
108.87
110.96
111.18
112.13
114.90
115.85
116.35
116.73
117.01
117.06
117.29
117.42
117.57
117.81
117.99
118.86
119.02
119.35
119.38
119.41
119.61
119.71
119.84
120.47
120.80
121.00
121.42
122.20
122.42
122.97
123.47
123.74
125.44
126.25
127.31
129.47
130.07
130.65
132.43
132.93
133.20
134.42
135.37
137.62
140.64
143.86
144.59
146.04
148.72
149.90
150.62
166.04
168.46
169.78
171.69
180.63
181.64
182.83
187.04
NAU3010a
Gh325
CIR388
HAU-SNP231
CIR026
HAU1567
HAU0761
JESPR302
NAU3287
JESPR308
JESPR157
MGHES29
NAU5128
NAU3158a
NAU2602
BNL1513
DPL0461
JESPR70
DPL0068
NAU3424c
HAU1739a
BNL2961
NAU3424b
MUSS250
NAU2439
GhPIP1-2-ss
GhPIP1-2-ds
HAU2738
NAU3201b
HAU1639b
BNL1521
NAU3721
HAU-SNP418
JESPR305
NAU3562
BNL3638
NAU3605a
HAU3247
JESPR78
HAU3293
NAU3499
HAU2015
NAU2169 MUCS113
BNL2616
NAU3708
NAU3804
NAU2434
HAU1846
NAU3071
DPL0191
TMB0072
HAU-InDel110a
TMB1639
HAU2640a
DPL0534
HAU0722
HAU1837
NAU6616
BNL3604
HAU2086a
NAU2619
Gh171
TMB0968
BNL2568
HAU3346a
Gh128
BNL2499
NAU2914a
BNL2655
NAU2240
HAU2522
NAU3988
TMB1182
DPL0231
NAU2731
BNL3084
NAU3207b
NAU6213
HAU1905
HAU1288b
NAU3954
JESPR127
MUSS255
Gh465
NAU2926
DPL0146
NAU6389
DPL0113
HAU1533
Gh573a
BNL252
BNL2582
STV025
NAU2230
MGHES22
NAU3667
NAU3771
NAU5379
HAU2631
Gh237a
CIR289
Gh146
JESPR307
NAU5335
Gh597
NAU3324b
NAU3904
NAU3668a
HAU1432
LMWHSP2-ds
BNL2835
HAU1628
TMB2386b

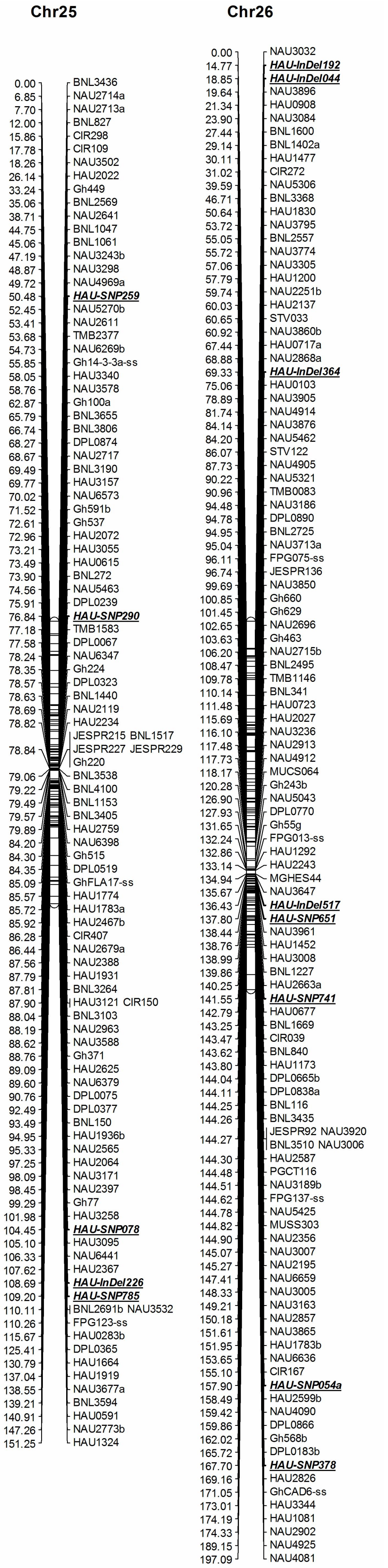
Chr25
0.00
6.85
7.70
12.00
15.86
17.78
18.26
26.14
33.24
35.06
38.71
44.75
45.06
47.19
48.87
49.72
50.48
52.45
53.41
53.68
54.73
55.85
58.05
58.76
62.87
65.79
66.74
68.27
68.67
69.49
69.77
70.02
71.52
72.61
72.96
73.21
73.49
73.90
74.56
75.91
76.84
77.18
77.58
78.24
78.35
78.57
78.63
78.69
78.82
78.84
79.06
79.22
79.49
79.57
79.89
84.20
84.30
84.35
85.09
85.57
85.72
85.92
86.28
86.44
87.56
87.79
87.81
87.90
88.04
88.19
88.62
88.76
89.09
89.60
90.76
92.49
93.49
94.95
95.33
97.25
98.09
98.45
99.29
101.98
104.45
105.10
106.33
107.62
108.69
109.20
110.11
110.26
115.67
125.41
130.79
137.04
138.55
139.21
140.91
147.26
151.25
BNL3436
NAU2714a
NAU2713a
BNL827
CIR298
CIR109
NAU3502
HAU2022
Gh449
BNL2569
NAU2641
BNL1047
BNL1061
NAU3243b
NAU3298
NAU4969a
HAU-SNP259
NAU5270b
NAU2611
TMB2377
NAU6269b
Gh14-3-3a-ss
HAU3340
NAU3578
Gh100a
BNL3655
BNL3806
DPL0874
NAU2717
BNL3190
HAU3157
NAU6573
Gh591b
Gh537
HAU2072
HAU3055
HAU0615
BNL272
NAU5463
DPL0239
HAU-SNP290
TMB1583
DPL0067
NAU6347
Gh224
DPL0323
BNL1440
NAU2119
HAU2234
JESPR215 BNL1517
JESPR227 JESPR229
Gh220
BNL3538
BNL4100
BNL1153
BNL3405
HAU2759
NAU6398
Gh515
DPL0519
GhFLA17-ss
HAU1774
HAU1783a
HAU2467b
CIR407
NAU2679a
NAU2388
HAU1931
BNL3264
HAU3121 CIR150
BNL3103
NAU2963
NAU3588
Gh371
HAU2625
NAU6379
DPL0075
DPL0377
BNL150
HAU1936b
NAU2565
HAU2064
NAU3171
NAU2397
Gh77
HAU3258
HAU-SNP078
HAU3095
NAU6441
HAU2367
HAU-InDel226
HAU-SNP785
BNL2691b NAU3532
FPG123-ss
HAU0283b
DPL0365
HAU1664
HAU1919
NAU3677a
BNL3594
HAU0591
NAU2773b
HAU1324
Chr26
0.00
14.77
18.85
19.64
21.34
23.90
27.44
29.14
30.11
31.02
39.59
46.71
50.64
53.72
55.05
55.72
57.06
57.79
59.74
60.03
60.65
60.92
67.44
68.88
69.33
75.06
78.89
81.74
84.14
84.20
86.07
87.73
90.22
90.96
94.48
94.78
94.95
95.04
96.11
96.74
99.69
100.85
101.45
102.65
103.63
106.20
108.47
109.78
110.14
111.48
115.69
116.10
117.48
117.73
118.17
120.28
126.90
127.93
131.65
132.24
132.86
133.14
134.94
135.67
136.43
137.80
138.44
138.76
138.99
139.86
140.25
141.55
142.79
143.25
143.47
143.62
143.80
144.04
144.11
144.25
144.26
144.27
144.30
144.48
144.51
144.62
144.78
144.82
144.90
145.07
145.27
147.41
148.33
149.21
150.18
151.61
151.95
153.65
155.10
157.90
158.49
159.42
159.86
162.02
165.72
167.70
169.16
171.05
173.01
174.19
174.33
189.15
197.09
NAU3032
HAU-InDel192
HAU-InDel044
NAU3896
HAU0908
NAU3084
BNL1600
BNL1402a
HAU1477
CIR272
NAU5306
BNL3368
HAU1830
NAU3795
BNL2557
NAU3774
NAU3305
HAU1200
NAU2251b
HAU2137
STV033
NAU3860b
HAU0717a
NAU2868a
HAU-InDel364
HAU0103
NAU3905
NAU4914
NAU3876
NAU5462
STV122
NAU4905
NAU5321
TMB0083
NAU3186
DPL0890
BNL2725
NAU3713a
FPG075-ss
JESPR136
NAU3850
Gh660
Gh629
NAU2696
Gh463
NAU2715b
BNL2495
TMB1146
BNL341
HAU0723
HAU2027
NAU3236
NAU2913
NAU4912
MUCS064
Gh243b
NAU5043
DPL0770
Gh55g
FPG013-ss
HAU1292
HAU2243
MGHES44
NAU3647
HAU-InDel517
HAU-SNP651
NAU3961
HAU1452
HAU3008
BNL1227
HAU2663a
HAU-SNP741
HAU0677
BNL1669
CIR039
BNL840
HAU1173
DPL0665b
DPL0838a
BNL116
BNL3435
JESPR92 NAU3920
BNL3510 NAU3006
HAU2587
PGCT116
NAU3189b
FPG137-ss
NAU5425
MUSS303
NAU2356
NAU3007
NAU2195
NAU6659
NAU3005
NAU3163
NAU2857
NAU3865
HAU1783b
NAU6636
CIR167
HAU-SNP054a
HAU2599b
NAU4090
DPL0866
Gh568b
DPL0183b
HAU-SNP378
HAU2826
GhCAD6-ss
HAU3344
HAU1081
NAU2902
NAU4925
NAU4081
